# Synergistic Cationic Shielding and Anionic Chemistry of Potassium Hydrogen Phthalate for Ultrastable Zn─I_2_ Full Batteries

**DOI:** 10.1002/adma.202411686

**Published:** 2024-10-22

**Authors:** Hao Fu, Shengyang Huang, Tian Wang, Jun Lu, Peixun Xiong, Kai Yao, Jin Suk Byun, Wenwu Li, Youngkwon Kim, Ho Seok Park

**Affiliations:** ^1^ School of Chemical Engineering Sungkyunkwan University 2066, Seobu‐ro, Jangan‐gu Suwon‐si, Gyeonggi‐do Republic of Korea; ^2^ Department of Electronics and Information Convergence Engineering Institute for Wearable Convergence Electronics Kyung Hee University Yongin‐si, Gyeonggi‐do 17104 Republic of Korea; ^3^ Institute of Energy and Climate Research Ma‐terials Synthesis and Processing (IEK‐1) For‐schungszentrum Jülich GmbH 52425 Jülich Germany; ^4^ Advanced Batteries Research Center Korea Electronics Technology Institute 25, Saenari‐ro Seongnam 13509 Republic of Korea; ^5^ SKKU Institute of Energy Science & Technology (SIEST) Sungkyunkwan University (SKKU) 2066, Seobu‐ro, Jangan‐gu Suwon Gyeonggi‐do 16419 Republic of Korea; ^6^ SKKU Advanced Institute of Nano Technology (SAINT) Sungkyunkwan University (SKKU) 2066, Seobu‐ro, Jangan‐gu Suwon Gyeonggi‐do 16419 Republic of Korea; ^7^ Department of Health Sciences and Technology Samsung Advanced Institute for Health Sciences and Technology (SAIHST) Sungkyunkwan University (SKKU) 2066, Seobu‐ro, Jangan‐gu Suwon Gyeonggi‐do 16419 Republic of Korea

**Keywords:** anions chemistry, electrostatic shielding cations, multifunctional additives, SEI, Zn metals

## Abstract

Electrolyte additives are investigated to resolve dendrite growth, hydrogen evolution reaction, and corrosion of Zn metal. In particular, the electrostatic shielding cationic strategy is considered an effective method to regulate deposition morphology. However, it is very difficult for such a simple cationic modification to avoid competitive hydrogen evolution reactions, corrosion, and interfacial pH fluctuations. Herein, multifunctional additives of potassium hydrogen phthalate (KHP) based on the synergistic design of cationic shielding and anionic chemistry for ultrastable Zn||I_2_ full batteries are demonstrated. K cations, acting as electrostatic shielding cations, constructed the smooth deposition morphology. HP anions can enter the first solvation shell of Zn^2+^ for the reduced activities of H_2_O, while they remain in the primary solvation shell and are finally involved in the formation of SEI, thus accelerating the charge transfer kinetics. Furthermore, by in situ monitoring the near‐surface pH of the Zn electrode, the KHP additives can effectively inhibit the accumulation of OH^−^ and the formation of by‐products. Consequently, the symmetric cells achieve a high stripping–plating reversibility of over 4500 and 2600 h at 1.0 and 5 mA cm^−2^, respectively. The Zn||I_2_ full cells deliver an ultralong term stability of over 1400 cycles with a high‐capacity retention of 78.5%.

## Graphical abstract

Multifunctional additives of potassium hydrogen phthalate (KHP) are rationally designed to achieve synergistic cationic shielding and anionic chemistry for ultrastable Zn||I_2_ full batteries. Electrostatic shielding K cations could construct the smooth deposition morphology effectively balancing the electric field distribution, while HP anions can reduce the activities of H_2_O and accelerate the charge transfer kinetics by the formation of SEI.

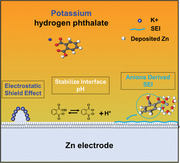

## 1 Introduction

Aqueous Zn‐based batteries are very attractive owing to their good safety, rich natural reserves, high theoretical capacity (820 mAh g^−1^ and 5855 mAh cm^−3^), and excellent processability for next‐generation energy storage systems.^[^ ^1^, ^2^, ^3^, ^4^ ^]^ However, the first solvation shell of Zn^2+^ with a high charge density possesses high covalency exhibiting a high overpotential of strong coulombic interaction between the Zn ion and the solvent. This strong interaction allows Zn^2+^ ions to show sluggish transport kinetics during the redox process and H_2_O in aqueous electrolytes to be decomposed into H_2_ through a parasitic hydrogen evolution reaction (HER) under a high overpotential.^[^ ^5^, ^6^, ^7^, ^8^, ^9^ ^]^ Furthermore, the insoluble by‐products (Zn_4_SO_4_(OH)_6_·*x*(H_2_O)) can be formed due to the pH fluctuation of the electrolyte localized at the electrode interface, which seriously deteriorates the life of the battery.^[^ ^10^, ^11^, ^12^, ^13^ ^]^ In particular, inevitable defects and bumps on the Zn surface lead to the construction of uneven electric fields for the non‐uniform Zn deposition.^[^ ^14^, ^15^, ^16^ ^]^ During the subsequent cycling process, these tip effects will be amplified to eventually form Zn dendrites, which results in a short circuit of the battery.

Electrolyte additive engineering has been known as a simple, effective, and low‐cost strategy to overcome the technical challenges in energy‐storing batteries.^[^ ^5^, ^17^, ^18^, ^19^, ^20^, ^21^, ^22^, ^23^, ^24^, ^25^, ^26^ ^]^ In particular, regulating the solvation structure of hydrated Zn^2+^ is an innovative way to promote a kinetic process and mitigate the competitive HERs and the formation of by‐products.^[^ ^27^, ^28^, ^29^, ^30^, ^31^, ^32^, ^33^ ^]^ Electrolyte additives such as dimethyl sulfoxide,^[^ ^34^ ^]^ organic gamma‐butyrolactone,^[^ ^35^ ^]^ 2,2,2‐trifluoroethano^[^ ^36^ ^]^ can effectively modify the solvation structure of Zn^2+^ due to the strong electron‐withdrawing effect, which reduces the bonding strength between Zn^2+^ and H_2_O molecules, thereby suppressing the HER and the formation of by‐products. In addition, some large molecular additives such as glucose molecules,^[^ ^37^ ^]^ graphitic carbon nitride quantum dots,^[^ ^38^ ^]^ and cetyltrimethyl ammonium bromide^[^ ^39^ ^]^ can be chemically bonded at the surface of Zn electrodes to avoid direct contact with H_2_O for anti‐HER capability. These strategies have demonstrated remarkable results in accelerating the reaction kinetics, diminishing the activity of parasitic water, and avoiding the HER and by‐products. During a long‐term cycling process, it is still difficult to avoid the challenges posed by dendrite growth, which becomes more serious at high current densities and depth of discharge. An electrostatic shield strategy based on cations has been exploited to inhibit this dendrite issue.^[^ ^40^, ^41^, ^42^, ^43^, ^44^, ^45^, ^46^, ^47^, ^48^ ^]^ Various cation additives such as Cs^+^,^[^ ^40^ ^]^ Ce^3+^,^[^ ^41^ ^]^ Li^+^,^[^ ^49^ ^]^ and Na^+[^ ^50^ ^]^ were employed to effectively modify the deposition morphology and to obtain a flat dendrite‐free metal anode. However, a simple cationic modification makes it very difficult to form a stable SEI and interface. Although the synergistic effect of multiple additives can solve the above problems at the same time,^[^ ^51^ ^]^ this will undoubtedly increase the complexity of the system and contradict the design goal of low cost.

Herein, we demonstrate a multifunctional electrolyte additive of potassium hydrogen phthalate (KHP) with a synergistic effect of the cations and anions on the reversible and stable Zn deposition for aqueous Zn metal batteries. Compared with other alkali metal ions, K^+^ ions achieve a larger ionic radius, smaller stroke radius, and low reduction potential. Therefore, K^+^ ions dissociated from KHP additives act as electrostatic shielding cations that can effectively balance the electric field distribution, smooth the deposition morphology, and inhibit the formation of dendrites. On the other hand, the anions entering the first shell of Zn hydrate form the contact ionic pairs (CIP), reducing the activity of parasitic H_2_O and accelerating the desolvation kinetics of Zn hydrates. The comprehensive analysis of the desolvation process elucidates that the KHP additives are reduced to form anion‐derived SEIs, which effectively inhibit corrosion and HER during the long‐term cycling, compensating the weak point of electrostatic shielding cation. Moreover, the KHP additive, used as the main component of the buffer solution, can effectively stabilize the interfacial pH fluctuations caused by the reduction process at the initial stage of the charge storage reaction. Benefiting from these advantages, the electrolyte with the synergistic effect of the cations and anions shows a greatly improved performance in both half and full cells. These findings provide new insights into the development and application of aqueous electrolyte chemistry.

## 2 Results and Discussion

### 2.1 Electrostatic Shielding Effect of K^+^ and Anion Chemistry of Hydrogen Phthalate Anions

**Figure 1:**
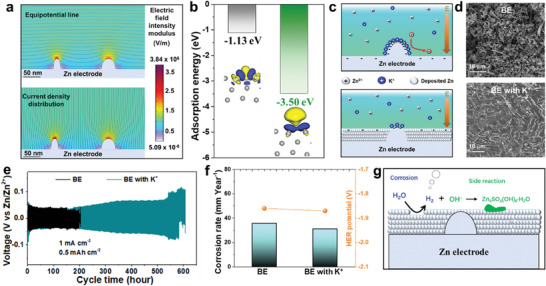
Electrostatic shielding effect of K+ in aqueous Zn system. a) Finite element simulation of actual Zn surface. b) Adsorption energy of Zn and K atoms on Zn (101) face. c) Working mechanism of the electrostatic shielding effect. d) Morphologies of cycled Zn with/without K^+^. e) Cycle performance of Zn symmetric cells with/without K^+^. f) Tafel and corrosion results calculated from Figures S5 and S6 (Supporting Information). g) Illustration of the disadvantages of the electrostatic shielding effect.

It has been known that commercial Zn foil is a polycrystalline material with inherent defects and protrusions formed during industrial production processes, which are difficult to remove by surface treatment, such as polishing or rolling. In order to confirm this, the actual surface condition of the Zn electrode, such as the equipotential line and current density distribution, was investigated using finite element simulation. As shown in **Figure** **1**a, the tips on the Zn metal surface are attributed to a locally concentrated equipotential and current density, which leads to the preferential adsorption of charged Zn^2+^ ions in aqueous electrolyte. After long‐term cycling, the formation of dendrites eventually occurs for cell failure (Figure S1, Supporting Information). Compared with Zn^2+^, K^+^ achieves a lower reduction potential (Table S1, Equations S1 and S2, Supporting Information) and a larger ionic radius, which results in showing a good shielding effect despite little amount. Furthermore, this is sufficiently stable under aqueous electrolyte environments without hydrolysis. More importantly, stokes radius is a measure of the diffusion dynamics of ions in solution, which is of importance for the electrostatic shielding effect of ions.^[^ ^52^, ^53^ ^]^

The Stokes radius (*r_s_*) of ions could be calculated by the following equation:

(1) $$r_{s}=\frac{k_{B}T}{6\pi \eta D}$$

where *k_B_*, *T*, *η*, and *D* are the Boltzmann constant, temperature, fluid viscosity, and diffusion coefficient of the system, respectively. Therefore, the Stokes radius of Li^+^ and Na^+^ could be calculated as 2.390 and 1.837 Å, respectively, larger than 1.253 Å of K^+^. The smaller stokes radius of K^+^ is beneficial for the ion transfer dynamics, thereby improving the shielding effect. These features allow K^+^ to be a promising candidate for electrostatic shield cation. DFT simulation was carried out to further investigate the adsorption behaviors of Zn^2+^ and K^+^ on the electrode surface. As shown in Figure 1b, Figure S2a, and Equation S3 (Supporting Information), the K^+^ shows higher adsorption energy than that of Zn^2+^ on different planes of Zn metals. The adsorbed K^+^ achieves a larger electron depletion region covering the surface (Figure S2b, Supporting Information), while the adsorbed Zn^2+^ with a limited electron depletion region is surrounded by an electron accumulation region. These results indicate that the K^+^ can be adsorbed on the Zn surface prior to Zn^2+^ providing a large electrostatic shielding region. Driven by the strong electric field, the K^+^ can be accumulated at the tips of the Zn electrode, but not reduced due to the lower reduction potential during a deposition process (Table S1, Supporting Information). Accordingly, the resulting positively charged region forces Zn^2+^ ions to be deposited at the flat region of the Zn electrode, thus avoiding the formation of dendrites (Figure 1c). In order to eliminate the influence of different anions, K_2_SO_4_ additives (same anions with ZnSO_4_) rather than KHP were employed to 2 M ZnSO_4_ (BE) to systematically explore the electrochemical performance. As shown in Figure 1e and Figure S3 (Supporting Information), the Zn symmetric cells with K^+^ achieve a longer lifespan from 300 to 600 h than ≈200 h of that without K^+^ additive at the concentration of K^+^ from 0.01 to 0.1 M. Furthermore, the cycled Zn electrode with K^+^ shows a flat and compact surface morphology, which is quite different from a mossy and dendritic structure without K^+^ (Figure 1d). These indicate that the K^+^ could regulate the deposition morphology of Zn ions to effectively inhibit the formation of dendrites.

In addition to the dendrite growth, corrosion and HER should be avoided for the reversible Zn deposition during a long‐term cycling process.^[^ ^3^, ^54^, ^55^ ^]^ The flat and smooth surface of Zn metal arising from (002) crystalline plane is beneficial for mitigating the occurrence of parasitic reactions.^[^ ^56^ ^]^ Therefore, XRD measurements are employed to investigate the orientation of crystalline Zn deposits by K^+^ additive. Unfortunately, as shown in Figure S4 (Supporting Information) and corresponding calculations (Equation S7, Supporting Information), the cycled Zn electrode with K^+^ additive achieves the same orientation as the original one (dominated by Zn (101) face) with a similar relative texture coefficients (29.64_(002)_, 27.69_(100)_ and 42.67_(101)_ for the electrode without K^+^; 33.02_(002)_, 21.85_(100)_ and 45.14_(101)_ for the electrode with K^+^, Table S2, Supporting Information),^[^ ^14^, ^57^ ^]^ which indicates that K^+^ cannot regulate the deposition crystal orientation of Zn. The electrochemical tests further indicate that irrespective of the existence of K^+^ in the electrolyte, two electrodes exhibit similar HER potentials (1.87 and 1.86 V with and without K^+^ at 10 mA cm^−2^, respectively) in linear sweep voltammetry (LSV) tests (Figure 1f; Figure S5, Supporting Information), and similar corrosion rates (31.0 and 35.7 mmol per year with and without K^+^, respectively) (Figure 1f; Figure S6, Equation S8, Table S3, Supporting Information). Moreover, the cycled Zn electrode with K^+^ shows obvious signals of by‐products (Zn_4_SO_4_(OH)_6_·*x*H_2_O) in XRD results (Figure S4, Supporting Information). Therefore, the K^+^ as electrostatic shielding cations can regulate the Zn deposition to improve the reversibility in aqueous electrolytes; however, this still faces the challenges of HER, corrosion, and by‐products even with K^+^ additive, especially under a long‐term cycling process (Figure 1g).

Based on the characteristics of anion chemistry,^[^ ^22^, ^58^ ^]^ the anions‐derived SEIs can be formed by introducing hydrogen phthalate (HP) anion participates in the solvation structure of aqueous electrolyte for the suppressed HER and corrosion. In order to confirm this, different amounts of KHP based on the solubility of KHP were added into pure ZnSO_4_ electrolytes. The concentrations of KHP are controlled into 0, 10, 50, and 100 mM, which are abbreviated as KHP‐0, KHP‐10, KHP‐50, and KHP‐100, respectively.

**Figure 2:**
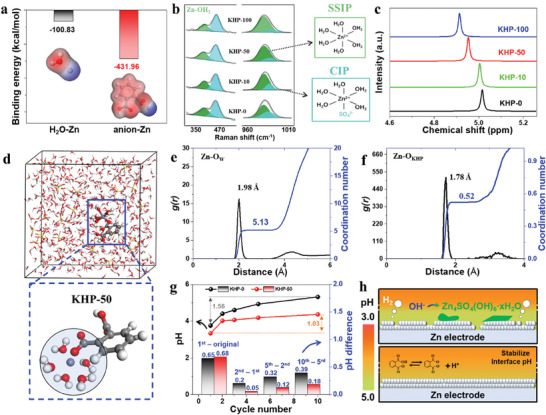
Solvation structure of KHP additive electrolytes. a) Binding energy of H_2_O and HP anion to Zn. b) Raman spectra and c) 1H NMR spectra of electrolytes with different numbers of additives. d) 3D snapshot of the KHP‐50 electrolyte and the enlarged solvation structure. RDFs and corresponding coordination number of e) Zn‐O_H2O_, f) Zn‐O_KHP_ collected from MD simulations in KHP‐50 electrolyte. g) pH values of the KHP‐0 and KHP‐50 electrolytes at different cycle states and corresponding pH difference calculations. h) Schematic illustration of surface condition in different electrolytes.

To investigate the effect of KHP additives on the solvation structure of Zn^2+^, DFT simulations are employed to investigate the interaction between Zn and solvents. As shown in **Figure** **2**a and Equation S4 (Supporting Information), the HP anion shows higher binding energy with Zn^2+^ (−431.96 kcal mol^−1^) than that of H_2_O with Zn^2+^ (−100.83 kcal mol^−1^), arising from the lone pair electrons from oxygen‐containing group in KHP, which indicates that the anions can be included in the solvation structure replacing H_2_O. Furthermore, the solvation structure is likely to be the ionic associations, as explained by the classic Eigen–Tamm mechanism.^[^ ^59^ ^]^ As shown in the Raman spectra (Figure 2b), the *v*‐SO_4_^2−^ can be resolved into two regions, solvent‐separated ion pair (SSIP, [Zn^2+^(H_2_O)_6_·SO_4_^2−^]) and contact ion pair (CIP, [Zn^2+^(H_2_O)_5_·OSO_3_^2−^]), respectively. Obviously, as the KHP amount increases, the peak intensity of CIP mode is weakened, while the peak intensity of SSIP increases from 65.6 to 89.6% (Table S4, Supporting Information). This observation implies that the abundant polar groups with electron‐donating effect (of carboxyl groups) in KHP are attributed to the strong interaction between HP anions and Zn^2+^, which contributes to weakening the binding strength between Zn^2+^ and SO_4_^2−^.^[^ ^59^, ^60^ ^]^ Furthermore, the Zn‐OH_2_ vibration at ≈ 390 cm^−1^ is suppressed from 41.5% (KHP‐0) to 30.7% (KHP‐100) with the increase of the KHP amount, indicating less H_2_O associated with Zn^2+^ by KHP additive (Table S4, Supporting Information). Correspondingly, the chemical shift of KHP‐0 at 5.01 ppm gradually shifts to lower fields at 4.98, 4.95, and 4.90 ppm for KHP‐10, KHP‐50, and KHP‐100, respectively, as shown in ^1^H nuclear magnetic resonance (NMR) spectra (Figure 2c). This finding means that the addition of KHP sheds out the bound H_2_O out of the primary solvation shell. The regulation of the solvation shell of Zn^2+^ by KHP is further supported by Fourier transform infrared (FTIR) spectra (Figure S7, Supporting Information). When the amount of KHP additive increases, SO_4_^2−^ stretching vibration peak is obviously a blue shift from 1083.8 to 1106.2 cm^−1^, which implies the impaired electrostatic coupling between Zn^2+^ and SO_4_^2−^. These results suggest that the KHP additive can effectively reconstruct the solvation structure in aqueous system. Among three samples of KHP‐10, KHP‐50, and KHP‐100, the KHP‐50 electrolyte was chosen for further investigations considering the best stripping–plating efficiency in Cu||Zn half cells (Figure S8, Supporting Information), the corresponding voltage profiles (Figure S9, Supporting Information), and the cycling reversibility in symmetric cells (Figure S10, Supporting Information).

Molecular dynamics (MD) simulations are performed to further clarify the solvation structures of the electrolytes. For the stabilized system, the KHP‐0 electrolyte shows the dominated structure of [Zn(H_2_O)_6_]^2+^ (Figure S11, Supporting Information), and the corresponding radial distribution function (RDF) of Zn‐O_H2O_ pairs estimates the distance of 2.01 Å with an average coordination number (CN) of 5.46. On the other hand, the KHP‐50 electrolyte shows the dominated structure of [Zn^2+^(5H_2_O·HP^−^)], and the decrease in the distance of 1.98 Å and the CN value of 5.13 derived from the RDF statistics of Zn‐O_H2O_ (Figure 2d,e). Moreover, RDF statistics of Zn‐O_KHP_ provide a shorter distance of 1.78 Å with an average CN of 0.52 than that of KHP‐0 system due to the strong interaction between polar function groups of KHP and Zn^2+^ (Figure 2f). These results elucidate that the anions are incorporated into the primary solvation shell of Zn^2+^, thereby reconstructing the solvation structure of [Zn(H_2_O)_6_]^2+^.

Along with the regulation of the solvation structure, KHP acts as the main component of the buffer solution to alleviate the change in the pH of the electrolyte to a certain extent, thus suppressing the formation of insoluble by‐products caused by the accumulation of OH^−^. In order to investigate the pH evolution at the electrode surface, the in situ cell is applied in different electrolytes during battery cycling (Figure S12, Supporting Information) according to reported references.^[^ ^11^, ^61^, ^62^ ^]^ At the initial step of the plating process, the pH values are rapidly raised (3.94 for KHP‐0; 3.49 for KHP‐50) and gradually stabilized (3.81 for KHP‐0; 3.38 for KHP‐50) in both electrolytes (Figure S13, Supporting Information), which may be caused by the directional movement of charged ions under the exerted electric field. After the systems reached equilibrium, the pH of the KHP‐0 increased by 1.56 from the initial to the 10th cycle (Figure 2g, Table S5, Supporting Information). For the KHP‐50 electrolyte, the pH increase is only 1.03. This suggests that the KHP‐50 can effectively stabilize the surface pH of the electrode to inhibit the accumulation of OH^−^, thus reducing the formation of irreversible by‐products of Zn_4_SO_4_(OH)_6_·*x*H_2_O (Figure 2h and Figure S14, Supporting Information). Moreover, the KHP‐50 electrolyte shows a slightly higher pH difference (0.68) than KHP‐0 electrolyte (0.65) after the 1st cycle, while in the subsequent cycles, these values are drastically dropped down to 0.05, 0.12, and 0.18, much lower than 0.20, 0.32, and 0.39 of KHP‐0 electrolyte after the 2nd, 5th and 10th cycles, respectively (Figure 2g). This trend is likely due to the fact that the pH of the KHP‐50 electrolyte is slightly lower than that of the KHP‐0 electrolyte, resulting in a higher pH difference of 0.68 at the initial cycle. Later, the participation of anions in the solvation structures enables to formation of a stable SEI during the initial few cycles, which results in inhibiting the direct contact between the electrolyte and the electrode, suppressing HER in long‐term cycling, and achieving a stable pH. This is further confirmed by LSV and in situ EIS measurements (Figures S15–S17, Supporting Information).

### 2.2 Desolvation Behavior of KHP Containing Electrolyte

**Figure 3:**
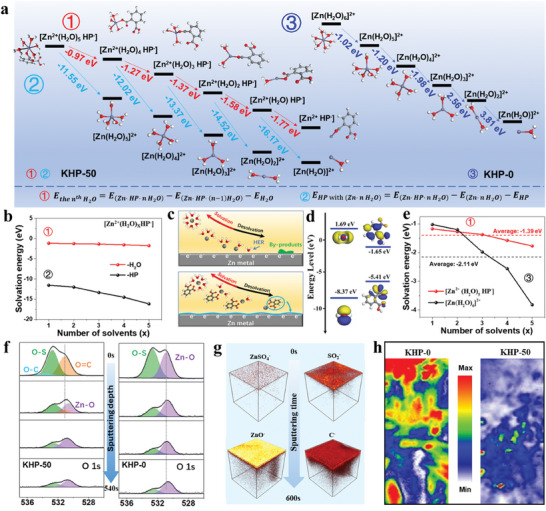
Desolvation process and the analysis of SEI. a) Different solvation steps and corresponding solvation energy in KHP‐0 and KHP‐50 electrolytes. b) The comparison of the solvation energy for KHP‐50 electrolytes in different solvation processes. c) Schematic illustration of the desolvation process of different electrolytes. d) Frontier molecular orbital energies calculation. e) Solvation energy in each step for KHP‐0 and KHP‐50 electrolytes. f) XPS spectral regions for O1s with a gradually increasing argon (Ar^+^) sputtering time. g) ToF‐SIMS 3D view of ZnSO_4_^−^, SO_2_^−^, C^−^ and ZnO^−^ with gradually increasing argon (Cs^+^) sputtering time for the electrolyte cycled in KHP‐50 electrolyte. h) Raman mapping of the electrode surface cycled in KHP‐0 and KHP‐50 electrolytes.

The formation of SEI is mainly attributed to the electrochemical decomposition of electrolyte components out of an electrochemical stability window during the initial cycling, which can improve the reversibility of Zn^2+^ plating–stripping for a long battery lifespan.^[^ ^58^, ^63^, ^64^ ^]^ In order to clarify the effect of anions on the formation of SEI in KHP‐50 electrolytes, the solvation process of different electrolytes was investigated by performing the DFT simulations. In this process, all possible solvation structures are considered, and the dissociation process of solvated Zn^2+^ is simulated one by one based on each stable configuration (Equations S5 and S6, Supporting Information). As shown in **Figure** **3**a (Tables S6 and S7, Supporting Information), the solvation process of KHP‐50 electrolytes could be summarised into 2 types. The solvation by H_2_O molecules (①, Table S6, Supporting Information) is less energetically favorable than that by HP anions (②, Table S7, Supporting Information) during the entire solvation number range from 1st to 5th steps (Figure 3b). This suggests that the HP anions remain solvated in the primary solvation shell of Zn^2+^ until the solvated H_2_O molecules are desolvated earlier than those. Subsequently, the partially desolvated Zn^2+^ coordinating with the HP anion, acquires electrons at the nucleation site under the electric field, and then, the latter is decomposed into a precursor of the final SEIs during the reduction of Zn^2+^ (Figure 3c). Although the large amount of free water in the aqueous electrolyte may also cause a reduction, the anion of KHP additive achieves a lower LUMO level compared with that of water, indicating an easier reduction of HP anions (Figure 3d). Consequently, anion‐derived SEIs could be constructed on the Zn surface, thereby preventing the electrode from direct contact with free water and guiding fast and uniform Zn^2+^ transportation. For the KHP‐0 electrolyte, the final SEI compositions would be dominated by Zn_x_SO_4_(OH)_6_ related materials due to the competition reaction of HER and Zn^2+^ reduction. We also compared the solvation energies of two electrolytes from 1st to 5th step. As shown in Figure 3e (Tables S6 and S8, Supporting Information), the KHP‐50 electrolyte shows a negatively lower average solvation energy (−1.39 eV) than that of the solvation structure in the KHP‐0 electrolyte (−2.11 eV). This solvation energy indicates a lower desolvation energy, which is consistent with the lower activation energy derived from the Arrhenius plots obtained from EIS tests under different temperatures (Figures S18 and S19 and Equation S9, Tables S9 and S10, Supporting Information).

In order to identify the chemical composition of SEIs, the Zn surface cycled in KHP‐0 and KHP‐50 electrolytes was analyzed using X‐ray photoelectron spectroscopy (XPS) depth sputtering. As shown in Figure 3f, the cycled Zn electrode in KHP‐0 electrolyte shows the O─Zn and O─S signals, which are preserved as sputtering time goes on. On the other hand, the decomposition of HP anions allows the surface of Zn electrode cycled in KHP‐50 electrolyte to achieve O─C and O═C signals. While increasing sputtering time, the O═C signal is gradually reduced and disappears, which is consistent with the C1s signals (Figure S20, Supporting Information). The ionic Zn species of by‐products are distributed from the surface to the inner of the electrode in KHP‐0, while the Zn electrode in KHP‐50 mainly consists of surface ionic Zn and inner metallic Zn (Figure S21, Supporting Information). As previously reported,^[^ ^65^, ^66^, ^67^ ^]^ the electrode surface in KHP‐0 is dominated by the accumulated by‐products due to the increase in a local pH value. On the other hand, the Zn electrode in KHP‐50 electrolyte is covered by the anion‐derived SEIs during the reduction process, due to the buffer function and the low LUMO of HP anions. The UV–vis spectroscopy demonstrates the reduction of anion concentration due to participation in SEI formation (Figure S22, Supporting Information). To further identify the composition of SEIs on the cycled Zn surface, the time‐of‐flight secondary‐ion mass spectrometry (ToF‐SIMS) and 2D Raman mapping results are collected in KHP‐0 and KHP‐50 electrolytes. As shown in Figure 3g and Figure S23 (Supporting Information), the species derived from Zn salt (or ZnSO_4_^−^, SO_2_^−^, ZnO^−^) appear with uneven distribution during the whole sputtering process in KHP‐0 electrolyte. By contrast, a complete anion‐derived SEI was formed on the electrode surface in KHP‐50, and only a few residual Zn salts at the surface (Figure S24, Table S11, Supporting Information). As shown in Raman mapping results (Figure 3h), the electrode cycled in KHP‐0 electrolyte reveals obvious signals of by‐products on a large area with uneven distributions. On the other hand, there is almost no signal of the by‐products on the surface of the electrode cycled in KHP‐50 electrolyte. Furthermore, the electrode cycled in KHP‐50 shows an amorphous structure on the surface with clear distributions of C and O elements, and the lattice patterns at the inner area from the inverse Fast Fourier Transform corresponding to the lattice distance of 0.249 nm, which is ascribed to Zn (002) plane, with a uniform Zn element distribution (Figure S25, Supporting Information). These findings further confirm that the electrode surface cycled in KHP‐50 is mainly composed of anion‐derived SEI‐containing carbon species and almost free from the by‐products.

### 2.3 Electrochemical Performance of Half/Full Cells With KHP Additives

**Figure 4:**
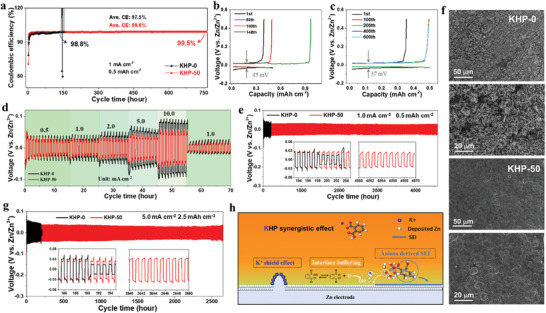
Electrochemical performance in half/symmetric cells. a) Coulombic efficiencies of half cells cycled in KHP‐0 and KHP‐50 electrolytes and corresponding voltage profiles for b) KHP‐0 and c) KHP‐50. d) Rate performance of Zn symmetrical cells in different current densities. Cycling performance of Zn symmetrical cells in KHP‐0 and KHP‐50 electrolytes under the current densities of e) 1.0 mA cm^−2^ (DOD: 0.34%) and g) 5.0 mA cm^−2^ (DOD: 1.7%). f) SEM images of electrode cycled in KHP‐0 and KHP‐50 electrolytes. h) Schematic illustration of the synergistic effect of the cations and anions of KHP‐50 electrolyte on the Zn surface.

As shown in **Figure** **4**a, the half cells cycled in KHP‐0 and KHP‐50 electrolytes achieve the average Coulombic efficiencies (CEs) of 97.5% and 99.0%, respectively, which were stabilized at 98.8% and 99.5%, respectively, at the end of the cycles. Moreover, the decomposition of thermodynamically unstable anions was accelerated at a high current density (Figure S26, Supporting Information), which promoted the formation of SEI. Thus, high average CEs over 99.8% were achieved at both 5.0 and 10.0 mA cm^−2^. For KHP‐0 electrolyte (Figure 4b), it shows a short lifespan of 148 h with a high average voltage hysteresis of 45 mV at the 100th cycle. On the other hand, KHP‐50 electrolyte shows a long lifespan of over 750 h with a low voltage hysteresis of 37 mV at the 100th cycle (Figure 4c). In addition, the electron transfer of the polar group allows KHP additives to show a lower adsorption energy of −2.4 eV than that of water molecules (−0.41 eV) on Zn (101) (Figures S27–S29, Supporting Information). This zincophilic property of KHP additives suppresses the direct contact between H_2_O and electrode surface reconstructing a water‐deficient electrical double layer for the inhibited corrosion of Zn electrode (Figure S30, Supporting Information). Moreover, KHP additives facilitate the interface transfer of Zn ions as verified by a low interfacial charge transfer resistance of 154.6 Ω (Figure S31 and Table S12, Supporting Information), higher ionic conductivity of 1.91×10^−2^ S cm^−1^ (Figure S32 and Equation S10, Supporting Information), and higher transference number of *t_Zn2+_* = 0.31 (Figure S33, Equation S11 and Table S13, Supporting Information). As shown in Figure 4d, KHP‐50 electrolyte achieves lower overpotentials (of 16.1, 18.5, 21.0, 28.2, and 39.8 mV) than those of KHP‐0 electrolyte (of 22.1, 24.5, 34.9, 51.5, and 79.1 mV) at different current densities of 0.5, 1.0, 2.0, 5.0, and 10.0 mA cm^−2^. Furthermore, the KHP‐50 electrolyte shows a super long reversibility over 4500 h at the current density of 1.0 mA cm^−2^ (Figure 4e), which is much higher than that of KHP‐0 electrolyte (≈200 h). Even at a high current density of 5.0 mA cm^−2^, KHP‐50 electrolyte could still deliver a high lifespan of over 2600 h (Figure 4g). Compared with other electrolyte additives published recently,^[^ ^68^, ^69^, ^70^, ^71^, ^72^, ^73^, ^74^ ^]^ our work shows better electrochemical performances in both overpotential and accumulative capacity in symmetric cells (Table S14, Supporting Information), which is attributed to the synergistic effect of excellent electrostatic shielding effect (K^+^) and anion chemistry.

For the practical application of Zn anodes, stripping–plating behavior in symmetric cells was evaluated at the depth of discharging (DOD) of 43% using a thin Zn electrode (11.7 mAh cm^−2^) with 20 µm thickness at the capacity of 5.0 mAh cm^−2^ (Equation S12, Supporting Information). Zn anode in KHP‐50 electrolyte exhibited a stable deposition profile for 350 h with a low overpotential of 76 mV. By contrast, Zn anode in the KHP‐0 electrolyte shows a short lifespan of 23 h with a high overpotential of 105 mV (Figure S34, Supporting Information). In addition, the electrode in KHP‐50 delivers a lower nucleation overpotential of 19 mV than 26 mV of KHP‐0 electrolyte (Figure S35, Supporting Information), indicating a low nucleation barrier and uniform growth of Zn. As shown in the SEM, in situ optical microscopy, and 3D confocal microscopies (Figure 4f; Figures S36–S38, Supporting Information), the electrode cycled in KHP‐50 electrolyte shows a dense and flat surface during the plating–stripping process with a low average roughness of 4.2 µm. The corresponding high‐resolution SEM and EDS mapping images exhibit a dense and even morphology with uniform HP^−^‐derived SEIs on the electrode surface (Figure S39 and Table S15, Supporting Information). On the other hand, the electrode cycled in KHP‐0 electrolyte exhibits an uneven and rough morphology on the surface after 30 min (Figure S36, Supporting Information). The corresponding SEM (Figure 4g; Figure S40, and Table S16, Supporting Information) and 3D confocal microscopy (Figure S38) images exhibit a loose and mossy structure, which is aggregated together at random with obvious dendrites characterization with a high average roughness (*R*_a_) of 25.8 µm. Furthermore, the adsorption energies of additives to different Zn planes play an important role in regulating the deposition crystal orientation.^[^ ^75^, ^76^ ^]^ Accordingly, the DFT calculations were employed to investigate whether KHP additive has the function of regulating the directional deposition of zinc ions into the specific crystalline plane. The adsorption energies of PH^−^ to Zn (100), (101), and (002) planes are −2.52, −2.40, and −2.65 eV, respectively (Figure S41, Supporting Information). The difference in adsorption energies among different planes is negligible, which implies no obvious selective adsorption of PH^−^ to the Zn electrode and a weak regulation of crystal orientation. The texture coefficient further calculated from XRD results also indicates that the electrode has no obvious orientation after cycling (Equation S7, Table S17, Supporting Information).

As shown in the chronoamperometric responses (Figure S42, Supporting Information), the current density in KHP‐50 electrolyte is stabilized in a very short period (within 30 s), indicating a very limited 2D diffusion process. The 3D diffusion‐dominated process after that is due to the synergistic result of cations with electrostatic shielding effect at tips and anions with interfacial buffering ability and stable SEI formation for the uniform and reversible Zn deposition (Figure 4h). On the other hand, the current density in KHP‐0 electrolytes gradually increases within 200s, corresponding to a long‐term 2D surface diffusion process of Zn ions. This 2D diffusion allows Zn ions to be nucleated and grown in favorable locations, such as the tips of the electrode surface with dense potential density, and eventually evolve into dendrites.

**Figure 5:**
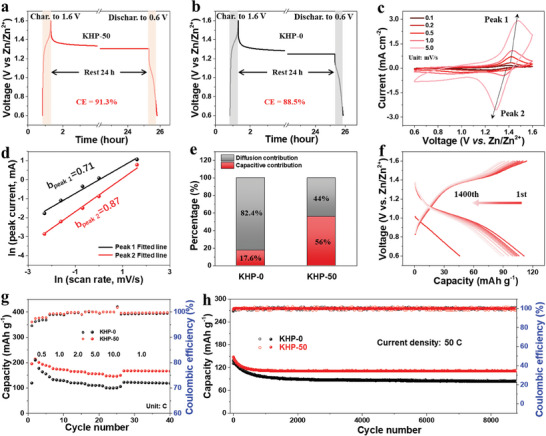
Electrochemical performance in Zn||I_2_ full cells. Standing voltage changes of a) KHP‐50 and b) KHP‐0 electrolyte. c) CV curves of KHP‐50 electrolyte at different scan rates. d) *b* values calculated from CV curves cycled in KHP‐50 electrolyte. e) Specific contribution of different electrolytes calculated from Figure S45 (Supporting Information). f) Voltage profiles of KHP‐50 under 2C rate. g) Rate performance and h) cycle performance of Zn─I_2_ full cells in KHP‐0 and KHP‐50 electrolytes.

The electrochemical performance of KHP‐0 and KHP‐50 electrolytes were further investigated by configuring Zn||I_2_ full cells. The carbon paper was employed as both host and substrate for the self‐standing I_2_ cathodes. Firstly, the self‐discharging of the full cell in different electrolytes was estimated to show the protection of SEI on the Zn electrode. As shown in **Figure** **5**a,b, after charging to 1.6 V, the full cells were rested for 24 h and then discharged to 0.6 V. The Zn||I_2_ cell in KHP‐50 electrolyte achieved a higher capacity retention of 91.3% than 88.5% of KHP‐0 electrolyte (Equation S13, Supporting Information), which is mainly attributed to the formation of KHP additive derived SEI and the inhibition of side reaction. As shown in the CV curves (Figure S43, Supporting Information), the full cell in the KHP‐50 electrolyte exhibited a low polarization of 72.8 mV due to the inhibition of side reactions and the formation of stable SEI with a small charge transfer resistance (68.29 Ω, Figure S44, and Table S18, Supporting Information). By contrast, the full cell in KHP‐0 electrolyte shows a large polarization of 142.0 mV with a large charge transfer resistance of 217.0 Ω, (Figure S44, Supporting Information).

The electrokinetic behavior of the electrolytes was further studied using CV at various scan rates. The peak current and scan rate follows a power‐law relationship (Equation S14, Supporting Information), in which the *b* value can determine the surface or diffusive electrochemistry of the system. As shown in Figure 5c,d (Figure S45, Supporting Information), the *b* values of the peaks from KHP‐50 are 0.71 and 0.87, respectively, >0.48 and 0.68 of KHP‐0 electrolyte, indicating a fast surface confined electrochemistry in KHP‐50 electrolyte. This surface‐confined electrochemistry in KHP‐50 electrolyte was further confirmed demonstrating a capacitive process of 56.0% higher than 17.6% in KHP‐0 as shown in Figure 5e (Figure S46, Equation S15, Supporting Information). Therefore, the full cell in KHP‐50 electrolyte delivers the specific capacities of 206.9, 179.6, 147.3, 110.7, and 95.1 mAh g^−1^ at the current rate of 0.1, 0.2, 0.5, 1.0, and 5.0 C, much higher than those of KHP‐0 electrolyte (202.5, 168.5, 127.1, 87.3, and 59.8 mAh g^−1^) as shown in Figure S47 (Supporting Information). When the current rate returned 0.2 C, the discharge capacity of KHP‐50 was recovered to the initial state and remained stable for a long cycle lifespan of over 400 h (Figure S48, Supporting Information). In order to investigate the effect of HP^−^ on the dissolution of polyiodides in full cells, the DFT calculations were employed on the interactions between HP^−^ and I^−^ (or I_2_ and I_3_^−^) species. As shown in Figure S49a (Supporting Information), the HP^−^ delivered much lower adsorption energy with I_2_ (−1.55 eV) than those with I^−^ (1.08 eV) and I_3_^−^ (0.88 eV) species, respectively, which indicates prior adsorption to I_2_. This preferential interaction leads to a shift in the equilibrium reaction left toward I_2_, thus reducing the concentration of polyiodides.^[^ ^77^ ^]^ The UV–vis spectroscopy results also show that the concentration of polyiodides decreased when the amount of HP^−^ increased (Figure S49b, Supporting Information), which implies the suppression of polyiodide dissolution by the HP^−^. In addition, the Zn electrode in KHP‐50 electrolyte shows a dense and smooth morphology after cycling in full cells (Figure S50, Supporting Information), while the electrolyte cycled in KHP‐0 electrolyte shows disordered morphology and agglomeration (Figure S50b, Supporting Information).

Therefore, as shown in Figure 5f and Figure S51 (Supporting Information), the full cell in KHP‐50 electrolyte exhibits a stable capacity of 86.7 mAh g^−1^ after 1400 cycles with a high‐capacity retention of 78.5% (Equation S13, Supporting Information) and a low capacity decay rate of 0.015 mAh g^−1^ per cycle (Equation S16, Supporting Information). By contrast, the specific capacity of the cell in KHP‐0 electrolyte was continuously decayed to ≈55.0 mAh g^−1^ with a low‐capacity retention of 52.4% and a high capacity drop rate of 0.032 mAh g^−1^ per cycle (Figure 5h and Figure S52, Supporting Information). Furthermore, the soft package battery in KHP‐50 can easily lighten LED lights (inset picture of Figure S51, Supporting Information), indicating a potential application prospect. In order to further highlight the electrochemical performance of the Zn─I_2_ batteries in KHP additive, the activated carbon was used as host materials to fabricate I_2_ cathodes through a carbon doctor‐blade casting method. As shown in Figure 5g, the full cell in KHP‐50 electrolyte delivered the specific capacities of 214.6, 176.5, 166.0, 155.8, and 143.3 mAh g^−1^ at the current rate of 0.5, 1.0, 2.0, 5.0 and 10.0 C, much greater than those of KHP‐0 electrolyte (210.5, 133.7, 120.8, 110.8, and 100.9 mAh g^−1^). In addition, the Zn─I_2_ full cell in KHP‐50 preserved a high capacity of 133.2 mAh g^−1^ with a high capacity retention of 85.0% after 2000 cycles at 10.0 C (Figure S53, Supporting Information). Even at 50.0 C (Figure 5h), the full cell still delivered the high capacity of 113.8 mAh g^−1^ over 8800 cycles. Therefore, the KHP‐50 additive shows superior electrochemical performances of specific capacity, current density, and capacity retention to previously reported works of electrolyte additives (Table S19, Supporting Information).

## 3 Conclusion

Based on the theoretical calculation and experiment, the effective regulation ability of K ion as an electrostatic shielding cation on the morphology of aqueous Zn ion deposition was first verified systematically. However, maintaining in an aqueous environment, the flat Zn surface is still challenged by slow kinetics, corrosion, hydrogen evolution, and other side reactions, which are obvious under long‐term cycling. A low‐cost, multi‐functional KHP additive was developed to realize a dendrite‐free anode with a long lifespan in aqueous Zn batteries. In particular, the electrostatic shielding K cations and the HP anion‐derived SEIs were expected to resolve the Zn dendrite growth, HER, and corrosion. The electrostatic shielding effect of K ions contributed to modifying the deposition morphology for the formation of dendrite‐free Zn. Moreover, the anions of KHP could enter the first solvation shell of hydrated Zn^2+^ to form contact ionic pairs, which suppress the parasitic reaction with H_2_O and accelerate the desolvation kinetics of hydrated Zn^2+^. Furthermore, as the main component of the buffer solution, KHP can alleviate the change in the pH of the solution caused by competitive HER, thus inhibiting the generation of insoluble by‐products. Therefore, symmetric cells with KHP additives showed a long cycling lifespan of over 4500 h under 1.0 mA cm^−2^ and even under a high depth of discharging state (44%), they can still maintain stability for over 350 h. Therefore, this work provides new insight into the rational design of emerging multifunctional additives based on the synergistic cations electric shielding effect and anions chemistry for long‐term stable Zn metal batteries.

## Conflict of Interest

The authors declare that they have no conflict of interest.

## Supporting information



Supporting Information

## Data Availability

The data that support the findings of this study are available from the corresponding author upon reasonable request.
