# Supplementary material for: Synergistic Cationic Shielding and Anionic Chemistry of Potassium Hydrogen Phthalate for Ultrastable Zn─I2 Full Batteries
Source: Adv Mater. 2024 Oct 22;37(3):2411686. doi: 10.1002/adma.202411686 (PMC11756037; doi:10.1002/adma.202411686)
Supplement: Supplementary file 1 — Supporting Information [file ADMA-37-2411686-s001.pdf]

ADVANCED MATERIALS

Supporting Information

for *Adv. Mater.*, DOI 10.1002/adma.202411686

Synergistic Cationic Shielding and Anionic Chemistry of Potassium Hydrogen Phthalate for Ultrastable Zn—I_2 Full Batteries

*Hao Fu, Shengyang Huang, Tian Wang, Jun Lu, Peixun Xiong, Kai Yao, Jin Suk Byun, Wenwu Li, Youngkwon Kim and Ho Seok Park**

Supporting Information

Synergistic Cationic Shielding and Anionic Chemistry of Potassium Hydrogen Phthalate for Ultrastable Zn-I$_2$ Full Batteries

Hao Fu,[1] *Shengyang Huang,*[1] *Tian Wang,*[2] *Jun Lu,*[1] *Peixun Xiong,*[1] *Kai Yao,*[3] *Jin Suk Byun,*[1] *Wenwu Li,*[1] *Youngkwon Kim,*[4] *Ho Seok Park* [1, 5, 6, 7, *]

[1] School of Chemical Engineering, Sungkyunkwan University, 2066 Seobu-Ro, Jangan-Gu, Suwon-si, Gyeonggi-do, Republic of Korea.

[2] Department of Electronics and Information Convergence Engineering, Institute for Wearable Conver-gence Electronics, Kyung Hee University, Yongin-si, Gyeonggi-do, 17104, Republic of Korea.

[3] Institute of Energy and Climate Research: Ma-terials Synthesis and Processing (IEK-1), For-schungszentrum Jülich GmbH, 52425, Jülich, Germany.

[4] Advanced Batteries Research Center, Korea Electronics Technology Institute, 25, Saenari-ro, Seongnam 13509, Republic of Korea.

[5] SKKU Institute of Energy Science & Technology (SIEST), Sungkyunkwan University (SKKU), 2066, Seobu-ro, Jangan-gu, Suwon, Gyeonggi-do 16419, Republic of Korea

[6] SKKU Advanced Institute of Nano Technology (SAINT), Sungkyunkwan University (SKKU), 2066, Seobu-ro, Jangan-gu, Suwon, Gyeonggi-do 16419, Republic of Korea.

[7] Department of Health Sciences and Technology, Samsung Advanced Institute for Health Sciences and Technology (SAIHST), Sungkyunkwan University (SKKU), 2066, Seobu-ro, Jangan-gu, Suwon, Gyeonggi-do 16419, Republic of Korea.

*E-mail: phs0727@skku.edu

I. Experimental Section

Materials: Zn foil with 250 μm thickness, Zinc sulfate ($ZnSO_4 \cdot 7H_2O$, 99.9%), and iodine (99.9%) were purchased from Alfa Aesar (ThermoFisher). Potassium hydrogen phthalate (99.95%) was purchased from Sigma-Aldrich. The carbon paper (Toray Carbon Paper 030) is purchased from The Fuel Cell Store. The high purity water was used to prepare aqueous electrolytes and solutions. The Zn foil with the thickness of 20 μm was prepared by a roller press.

Preparation of electrolytes and cathode: The electrolytes with different additive concentration (10, 50, 100 mM) was finished by adding different amount of potassium hydrogen phthalate into aqueous Zinc sulfate solutions. The 2M Zinc sulfate solutions with 0 mM potassium hydrogen phthalate additive are used to be the controlled sample. The iodine with carbon paper with different ratios was seal and then heated to 80 ^{o}C for 12 hours. The iodine cathode was encapsulated in the carbon paper and active carbon by a melt-diffusion method.[1, 2] More specifically, the iodine with carbon paper with the ratio of 1:1 was sealed and then heated to 80 ^{o}C for 12 hours to prepare the free standing I_2 cathode. The iodine with YP-50 active carbon in the ratio of 1:1 was seal and then heated to 80 ^{o}C for 12 hours. The electrode were prepared by the doctor-blade casting method on carbon paper by mixing the active material (80 wt%), carbon black (10 wt%), and sodium alginate (10 wt%) in water solution to form a slurry. The mass loading of the I_2 is controlled from 1.0 to 2.0 mg cm^{-2}. The voltage window of 0.6-1.6 V (vs. Zn^{2+}/Zn) is employed in the full cells for the electrochemical performance.

Structural and chemical characterization: The crystalline structures were obtained by X-ray diffraction (XRD) with Cu Kα radiation (λ = 0.154 nm) and a scanning rate of 3° min^{-1}. The Raman spectra and 2D raman mapping was obtained using NTMDT2 confocal Raman spectrometer (NDMDT) with a laser beam wavelength of 532 nm. The FT-IR Spectrometer

(IRTracer-100, Shimadzu, Japan) was employed for Fourier-transform infrared (FT-IR) spectra measurements. The ultraviolet–visible spectroscopy (UV-vis) was tested on a double beam UV-Visible spectrophotometer (JASCO V-750). To ensure the accuracy of the results, all of the tested electrolytes were diluted at the same multiple in advance. The ^{1}H nuclear magnetic resonance (NMR) spectra were recorded on a 700 MHz spectrometer (Bruker, AVANCEIII700) and the samples were prepared by mixing 0.4 mL electrolyte with 0.1 mL D_2O with internal standard. The Smartzoom 5 (Zeiss) optical microscopy was employed for the in-situ observation of the Zn deposition behaviour. The XPS was conducted using a Thermo-Scientific ESCALAB 250Xi. The TOF-SIMS-5 (ION-TOF, Germany) with Cs+ source was employed to obtain the Time-of flight secondary-ion mass spectrometry (ToF-SIMS) at various sputtering time (0-600s). The morphologies were obtained by field emission scanning electron microscopy (FESEM, Hitachi S-4800). 3D images of the Zn electrodes were collected using scanning confocal laser microscopy (Keyence, VK-2000).

Electrochemical measurements: The electrochemical characterization including Zn||Cu half-cells, Zn symmetric cells, and Zn||I_2 full cells were obtained using CR2032-type coin cells. The galvanostatic charge/discharge performance, rate performance, voltage profiles, and Coulombic efficiency of different cells were obtained using the WonAtech WBCS3000L automatic battery test system and Neware CT-4008Tn-5V50mA Battery Tester. Electrochemical impedance spectroscopy (EIS), linear sweep voltammetry (LSV), chronoamperometry (CA), and cyclic voltammetry (CV) tests were conducted using a Bio-logic VMP-3 electrochemical station. EIS was conducted over a frequency range of 300 KHz to 0.1 Hz with an amplitude of 10 mV using a Bio-logic VMP-3 electrochemical station. The corresponding Tafel and LSV plots were obtained using a three-electrode system comprising a platinum as the counter electrode, a Ag/AgCl as the reference electrode, and a Zn foil as the

working electrode. Nucleation overpotential was measured by the a three-electrode system including a Ti foil electrode, a Ag/AgCl electrode, and a Zn foil as the working electrode, counter electrode, and the reference electrode, respectively.

II. Calculation methods

(1) Standard electrode potential

$$K^{+} + e^{-} = K\ (s) \qquad E^{\Theta} = -2.931\ V \tag{S1}$$

$$Zn^{2+} + 2e^{-} = Zn\ (s) \qquad E^{\Theta} = -0.7618\ V \tag{S2}$$

(2) Finite Element Analysis Method

The simplified 2D model consists of two electrodes and an intermediate electrolyte region. This simulation uses the electric field intensity and currents to present the specific condition of electrode surface. The variable for the normal current density defines the mesh velocity. The electrode features several protrusions, which represent tips of the Zn surface.

(3) Density functional theory calculation

The first-principles calculations based on density functional theory (DFT) were conducted using the Castep module of Materials Studio by generalized gradient approximation (GGA) and Perdew Burke Ernzerhof (PBE) exchange-correlation functional. Due to the practical Zn foil is dominated by (101) crystal face, so the crystaline structure of Zn (101) was employed to conduct a 3x3x1 supercell with a 15 Å vacuum layer. After convergence testing, the cutoff energy was selected to be 400 eV, and the convergence tolerance of the geometry optimization were set to 1.0×10^{-5} eV/atom for energy, 2.0×10^{-2} eV/Å for maximum force, and 1.0×10^{-3} Å for maximum displacement.

The adsorption energies of Zn(101) with K and Zn atoms were calculated by the following equation:[3]

$$\boldsymbol{E_{ad} = E_{s} - E_{Zn(101)} - E_{x}} \tag{S3}$$

where $\boldsymbol{E_{ad}}$, $\boldsymbol{E_{Zn(101)}}$ and $\boldsymbol{E_x}$ represent the total energy of the zinc with atom, the energy of zinc, and the energy of adsobed atom, respectively.

The binding energies of Zn with H_2O and additive were performed with B3LYP method in the Dmol3 module of Materials Studio, according to the following equation:

$$\boldsymbol{E_b = E_{total} - E_{Zn} - E_a} \qquad (S4)$$

where $\boldsymbol{E_{total}}$ is the structure total energy, $\boldsymbol{E_{Zn}}$ is the energy of Zn ion, and $\boldsymbol{E_x}$ is the energy of different molecule fragments (X = H_2O, additive). The convergence tolerances of the systems were set to 1.0 × 10^{-5} eV/atom for energy, 0.002 Ha/Å for maximum force, and 5.0×10^{-3} Å for maximum displacement.

Based on the solvation structure of KHP-50 electrolyte ([Zn· HP· $5H_2O$]), after considering all the possible solvation structures, the dissociation process of solvated Zn^{2+} could be summarised into 2 types. The first one only considered the desolvation process of the solvent (H_2O), which could be calculated as following equation[4]:

$$\boldsymbol{E_{(the\ n^{th}\ H_2O)} = E_{(Zn\cdot\ HP\cdot\ n\,H_2O)} - E_{(Zn\cdot\ HP\cdot\ (n-1)H_2O)} - E_{H_2O}} \qquad (S5)$$

where $\boldsymbol{E_{(the\ nth\ H2O)}}$ is the solvation energy of the nth solvent molecular, $\boldsymbol{E_{(Zn\cdot\ HP\cdot\ n\ H2O)}}$ is the total enery of the solvated sturcture, $\boldsymbol{E_{(Zn\cdot\ HP\cdot\ (n-1)H2O)}}$ is the energy of the structure without the nth solvent molecular, $\boldsymbol{E_{H2O}}$ is the energy of the nth solvent.

The second one considered the desolvation process of the additive (HP anion) in the solvation structures with different number of solvent molecular, which could be calculated as following equation[4]:

$$\boldsymbol{E_{HP} = E_{(Zn\cdot\ HP\cdot\ n\,H_2O)} - E_{(Zn\cdot\ n\,H_2O)} - E_{HP}} \qquad (S6)$$

where $\boldsymbol{E_{HP}}$ is the solvation energy of the additive, $\boldsymbol{E_{(Zn\cdot\ HP\cdot\ n\ H2O)}}$ is the total enery of the solvated sturcture, $\boldsymbol{E_{(Zn\cdot\ n\ H2O)}}$ is the energy of the solvation structure without anion, $\boldsymbol{E_{HP}}$ is the energy of the solvated anion.

The molecular dynamics (MD) was conducted by the forcite analysis of Materials Studio using the same atomic ratio of KHP-0 and KHP-50 electrolytes. The interactions between molecules were described using the COMPASS III force field. The systems was firstly relaxed by microcanonical ensemble (NVE) under 300 K for 500 ps, then equilibrium density by constant-pressure, constant-enthalpy ensemble (NPH), and canonical ensemble (NVT) for 1.0 ns at 300 K and 1 atmospheric pressure for energy minimization of the initial structure. Finally, the system was conducted for 7.0 ns at NVT with a time step of 1.0 fs. The results were obtained from the last 5.0 ns of the NVT process. The determine of the final solvation structure and statistical summary of radial distribution functions (RDFs) were all based on the final structures by using the Forcite analysis tool.

(4) Relative texture coefficiency calculation

The relative texture coefficient was calculated by the following equation:

$$\boldsymbol{RTC_{(hkl)}} = \frac{\boldsymbol{I_{1(hkl)}/I_{S(hkl)}}}{\sum(\boldsymbol{I_{1(hkl)}/I_{S(hkl)}})} * \mathbf{100} \tag{S7}$$

where $\boldsymbol{I_{1(hkl)}}$ is the intensity from the reacted sample, and $\boldsymbol{I_{S(hkl)}}$ is the intensity from the standard sample.

(5) Corrosion rate calculation

The corrosion rate (C_R) was calculated by the following equation:

$$\boldsymbol{C_R} = \frac{\boldsymbol{I_{corr} \times K \times EW}}{\boldsymbol{D \times A}} \tag{S8}$$

where $\boldsymbol{K}$ is a constant (3270 mol A^{-1}), $\boldsymbol{I_{corr}}$ is the corrosion current density, $\boldsymbol{EW}$ is the mole equivalent of Zn, $\boldsymbol{D}$ is the density of Zn, and $\boldsymbol{A}$ is the sample area.

(6) The activation energy calculation

The activation energy ($\boldsymbol{E_a}$) was calculated from the EIS of the symmetric cells under different temperatures according to the follow equation:

$$\frac{1}{R_{ct}} = Ae^{-\frac{E_a}{RT}} \tag{S9}$$

where R_{ct}, E_a, R, T, and A are the charge transfer resistance through the electrode/electrolyte interface, activation energy, gas constant, temperature in Kelvin, and the frequency factor constant, respectively.

(7) Ionic conductivity calculation

The ionic conductivity was tested using two blocking electrodes on Ti foils. This was calculated using the following equation at room temperature:

$$\sigma_i = \frac{L}{R_c \cdot S} \tag{S10}$$

where L, S, Rc are the thickness of the separator, the contact area, and the resistance, respectively.

(8) Transference number calculation

The transference number of Zn^{2+} was obtained by the EIS measurements in a symmetrical battery before and after the chronoamperometry (CA) tests by the following equation:

$$t_{Zn^{2+}} = \frac{I_s(\Delta V - I_0 R_0)}{I_0(\Delta V - I_s R_S)} \tag{S11}$$

where I_s and R_s are the steady state current and resistance, I_0 and R_0 are the initial current and resistance, and ΔV is the applied voltage (here, 20 mV) polarization, respectively.

(9) Depth of discharging calculation

The depth of discharging calculation were evaluated based on following equation:

$$DOD = \frac{C_{Dis}}{S_{ele} * THK_{ele} * C_{theory} * \rho_{Zn}} * 100 \tag{S12}$$

where C_{dis}, S_{ele}, THK_{ele}, C_{theory}, and ρ_{Zn} are the specific capacity of discharging, electrode surface area, electrode thickness, electrode theorical capacity, and electrode density, respectively.

(10) Capacity retention calculation

The capacity retention of the full batteries at long cycle and rest 24 hours were evaluated based on same equation but different definitions:

$$\boldsymbol{C_R = \frac{C_L}{C_I}\ X\ 100\%} \quad \text{(S13)}$$

For Capacity retention at long cycle, where $\boldsymbol{C_R}$ is the capacity retention, $\boldsymbol{C_L}$ is the capacity in the last cycle, and $\boldsymbol{C_I}$ is the capacity in the first cycle.

For Capacity retention during rest for 24 hours, where $\boldsymbol{C_R}$ is the capacity retention, $\boldsymbol{C_L}$ is the discharge capacity after rest, and $\boldsymbol{C_I}$ is the charge capacity before rest.

(11) Kinetic analysis

The capacity contribution could be calculated with following equation:

$$\boldsymbol{i = av^b} \quad \text{(S14)}$$

$$\boldsymbol{i = k_1 v + k_2 v^{1/2}} \quad \text{(S15)}$$

where *i* is current, *v* is scan rate, and *b* is determined by the slope of the plot of *ln i* and *ln v*. and k_1v and $k_2v^{1/2}$ are the current contributions of the capacitance-controlled process and diffusion-controlled process, respectively.

(12) Capacity drop rate

The capacity retention of the full batteries was evaluated based on their difference in capacity between the first and end cycles, according to the following equation:

$$\boldsymbol{C_{DR} = \frac{C_I - C_L}{CN}} \quad \text{(S16)}$$

where $\boldsymbol{C_{DR}}$ is the capacity drop rate, $\boldsymbol{C_L}$ is the capacity in the last cycle, $\boldsymbol{C_I}$ is the capacity in the first cycle, and ***CN*** is the total cycle numbers.

III. Supplementary figures

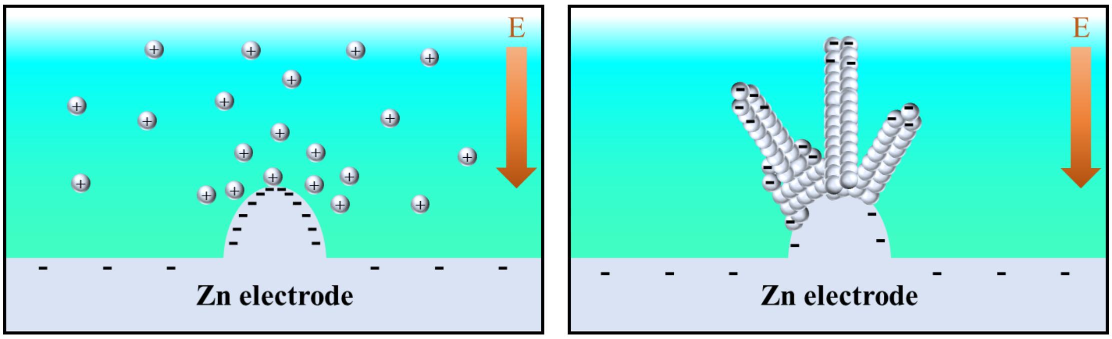

Figure S1. Illustrition of tip effects in aqueous Zn batteries.

Note for Figure S1: The surface tip on Zn electrode will lead a concentrated current density and equipotential, which will induce a porrior deposition of Zn ions at the tip and gradually lead the dendrite growth causing the battery failure.

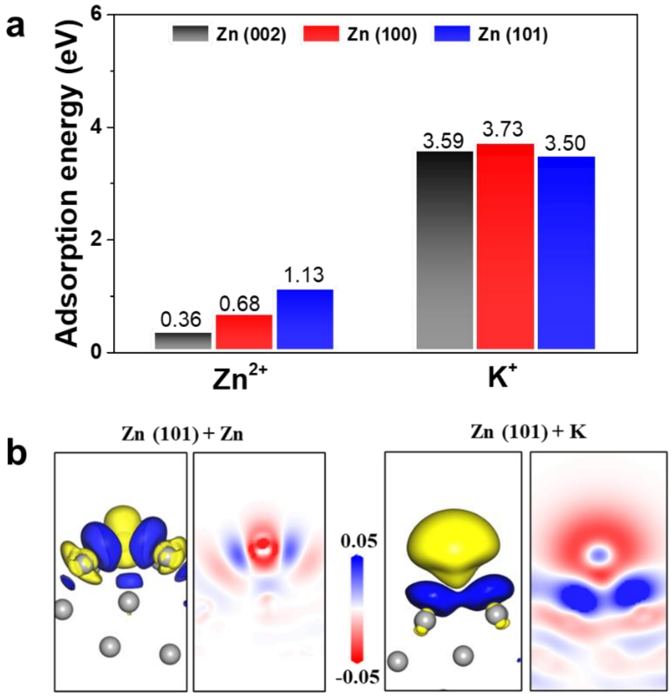

Figure S2. (a) adsorption energies of Zn^{2+} and K^{+} on different crystal planes of Zn. (b) Electron density differences of different adsorbates on Zn (101) face.

Note for Figure S2: Compared with the adsorption energies of Zn^{2+} on different crystal planes of Zn electrode, the adsorption energies of K^{+} on Zn different planes always show negatively higher values, which provides an effective basis for the electrostatic shielding effect of potassium ions on Zn electrode.

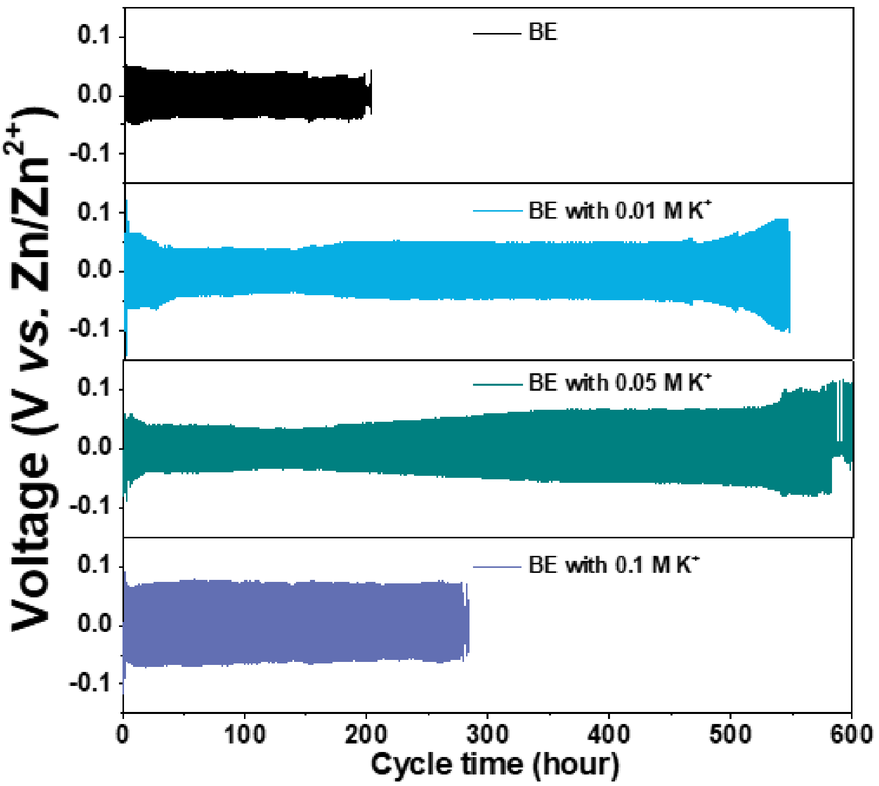

Figure S3. The electrochemical performance of symmetric cells with different electrolytes.

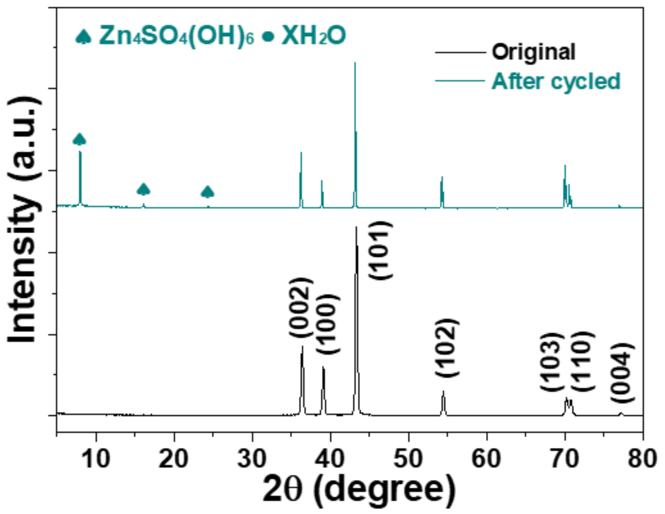

Figure S4. XRD results of electrodes before and after cycling with K+.

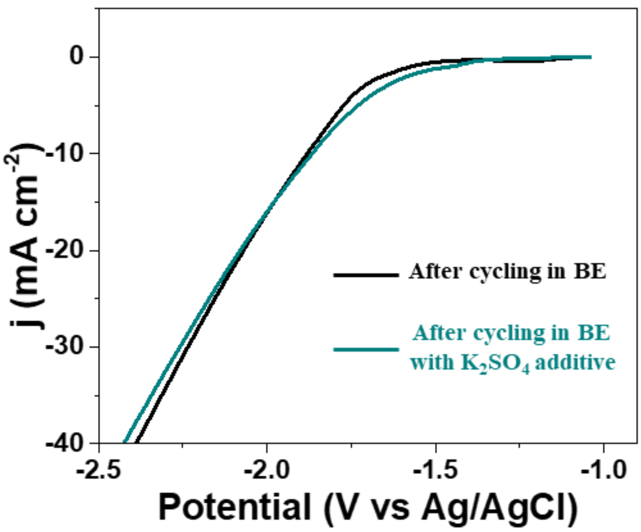

Figure S5. Linear sweep voltammetry measurements of the electrodes after the 10th cycle.

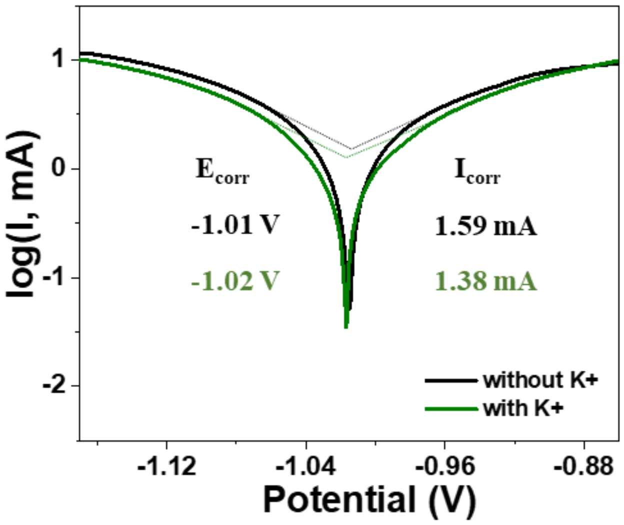

Figure S6. Tafel plots (letf) and corresponding calculations (right) of the electrodes tested in BE and BE with K_2SO_4 additive.

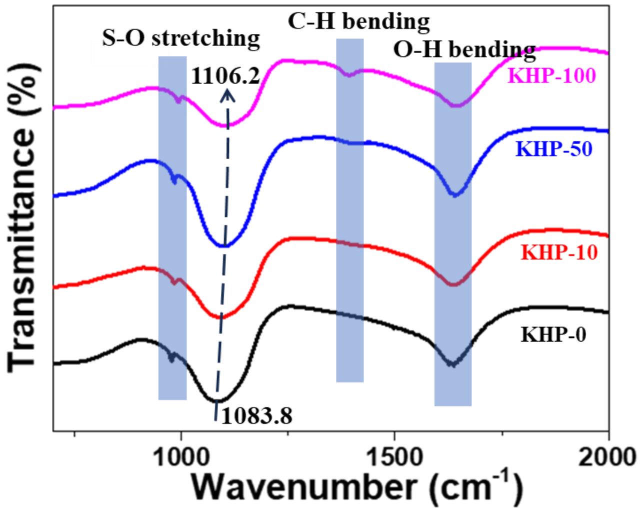

Figure S7. FT-IR spectra of electrolytes with different amount of additives.

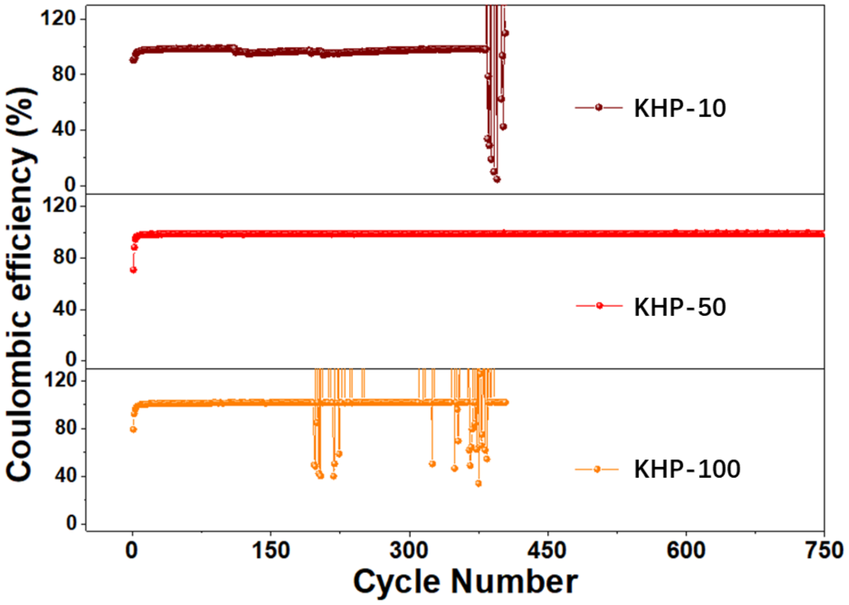

Figure S8. Coulombic efficiencies of Zn||Cu half cells with different amount of additives under 1.0 mA cm^{-2} with the capacity of 0.5 mAh cm^{-2}.

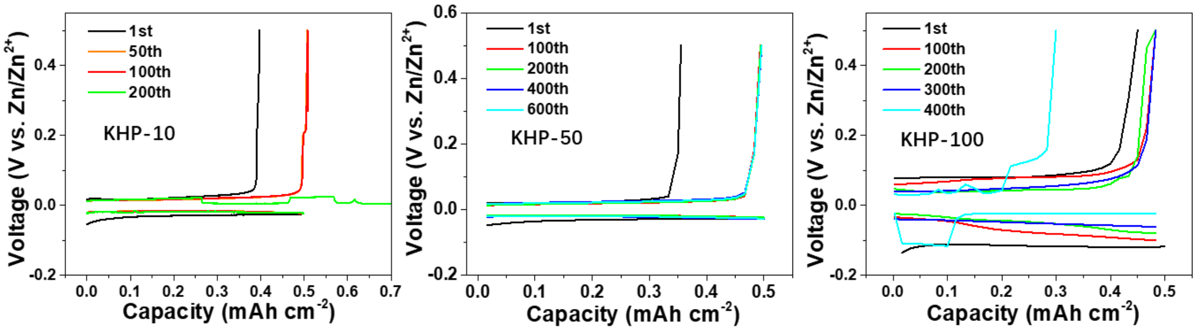

Figure S9. Galvanostatic charge–discharge profiles of Zn||Cu half cells with different amount of additives under 1.0 mA cm^{-2} with the capacity of 0.5 mAh cm^{-2}.

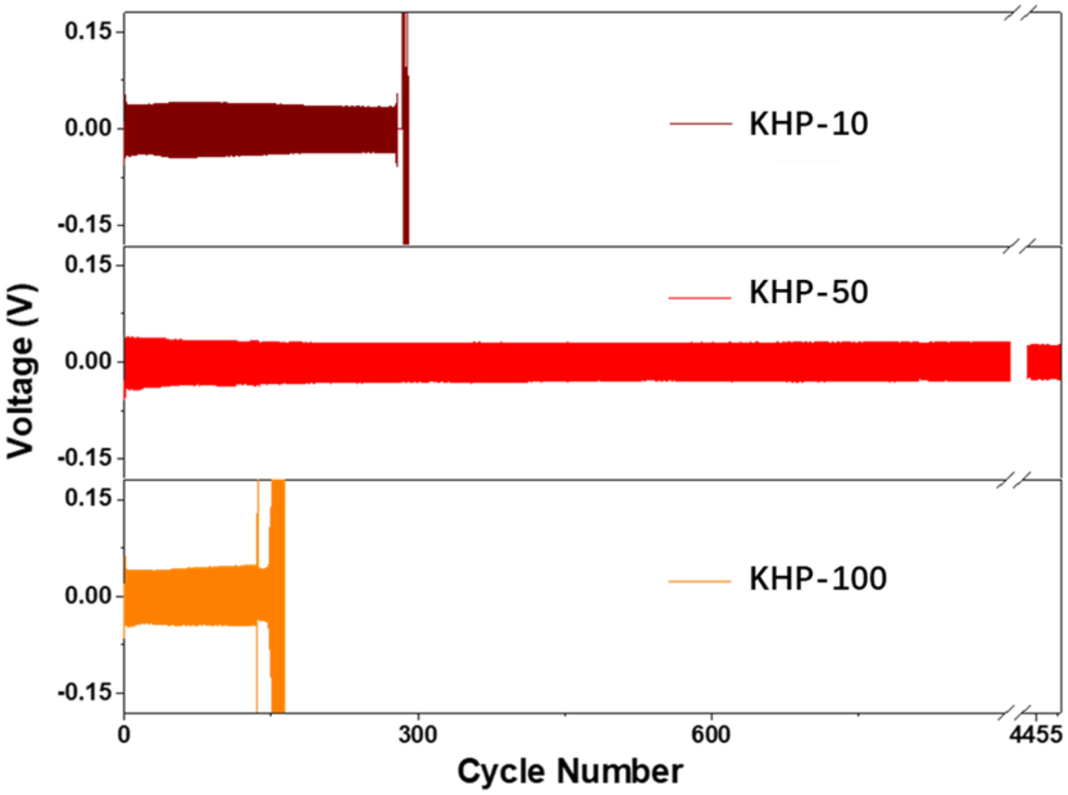

Figure S10. Galvanostatic charge–discharge profiles of Zn||Zn symmetric cells with different amount of additives under 1.0 mA cm^{-2} with the capacity of 0.5 mAh cm^{-2}.

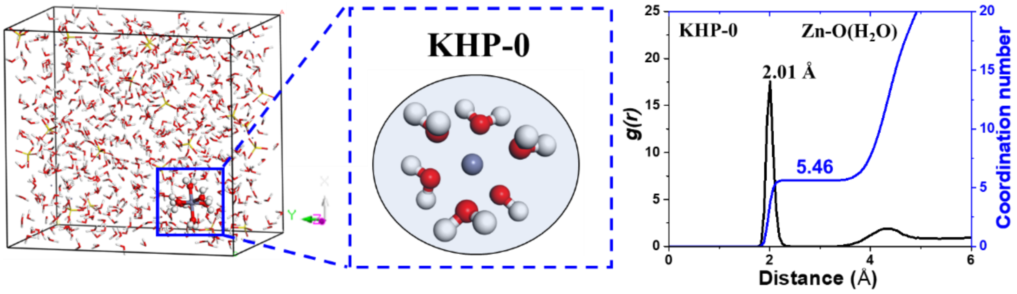

Figure S11. 3D snapshot of the KHP-0 electrolyte by MD simulations, representative Zn^{2+}-solvation structure, and corresponding RDF for Zn-O_{H2O} and average coordination number of Zn^{2+} collected from MD simulations.

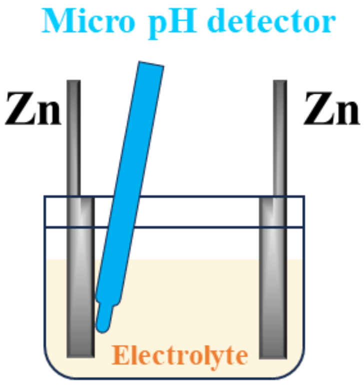

Figure S12. The design of *in-situ* device for pH detection during cycling.

***Note for Figure S12*:** The standard Q size (L:52.5 mm, H: 45.0 mm, W:12.5 mm) quartz glass (uv-vis cuvette) is employed as the container of the device. To obtain pH information at the near surface, a micro detector was employed to minimize the distance between the pH detector and the working electrode, and it was in close contact with the zinc electrode during the entire measurement process to ensure accurate pH information near the interface. The pH value is recorded on the working electrode (every 5s) during the whole cycling process under a current density of 5.0 mA cm^{-2}.

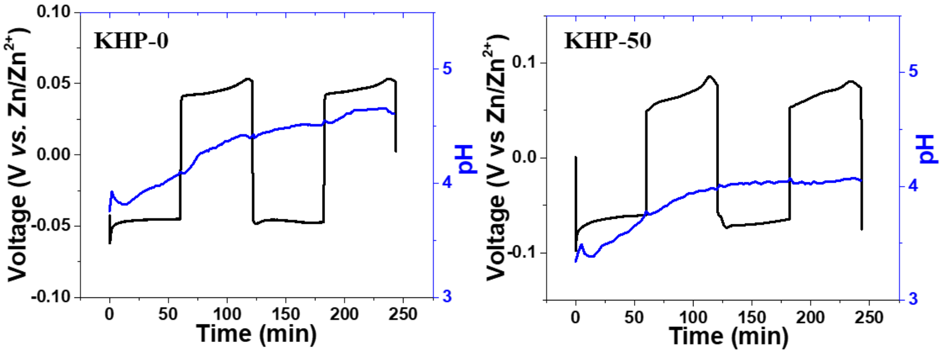

Figure S13. The *in-situ* pH measurements of KHP-0 and KHP-50 electrolytes under 2.0 mA cm^{-2}.

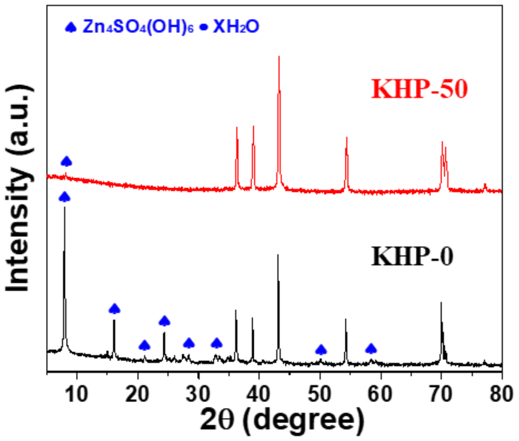

Figure S14. The XRD results of the electrodes cycled in KHP-0 and KHP-50 electrolytes.

Note for Figure S14: Benefiting from the buffer function of KHP additives that can inhibit the pH fluctuations and accumulation of OH^-, the electrode cycled in KHP-50 has almost no signals for by-products in the XRD results. In contrast , the electrode cycled in KHP-0 electrolyte shows obvious signals of by-product ($Zn_4SO_4(OH)_6 \cdot XH_2O$).

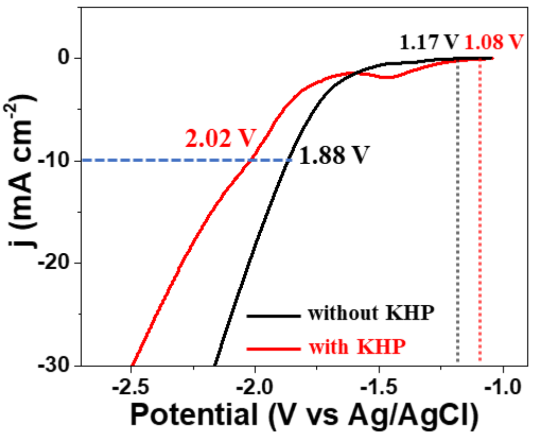

Figure S15. Linear sweep voltammetry of original electrodes with/without KHP additives.

Note for Figure S15: For the origional electrodes, the electrolyte with KHP additives shows higher HER onset potential of -1.08 V than that of the electrolyte without KHP additives, which is attributed to the slight lower pH of KHP-50 electrolyte. And an additional current signal could be found for KHP-50 electrolyte at ~1.48 V, which is possibly attributed to the decomposition of KHP additives. After that the KHP-50 shows a lower potential (2.02 V) than that of KHP-0 (1.88 V) at 10 mA cm^{-2}.

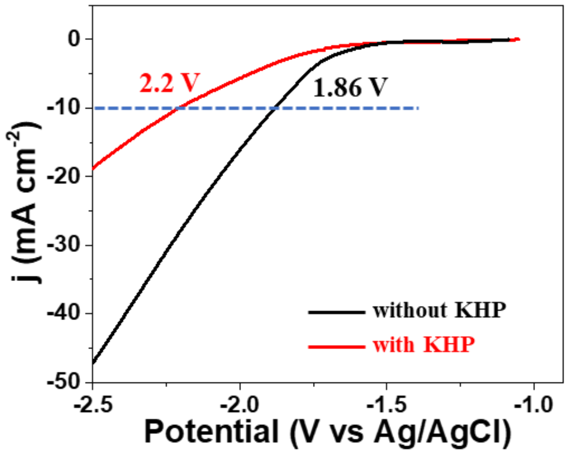

Figure S16. Linear sweep voltammetry of electrodes after the 10th deposition with/without KHP additives.

Note for Figure S16: For the electrodes after the 10th deposition, the electrolyte with additives shows a large difference of the potential (0.34 V) at 10 mA cm^{-2}. This suggests that the KHP

derived SEI could effective inhibit the direct contact between the electrolyte and the electrode thus reducing HER.

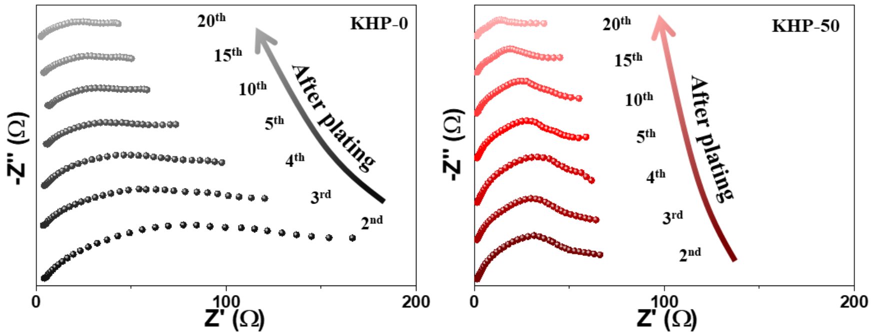

Figure S17. In-situ EIS measurements of KHP-0 and KHP-50 electrolytes from the 2nd to the 20th cycle.

Note for Figure S17: The in-situ EIS measurements of Zn symmetric cells are performed to investigate the stability of the interface formed in different electrolytes. Benefiting from the formation of anion derived SEI, the KHP-50 electrolyte delivers a rapid stabilization of Rct during the first few cycles, indicating a fast and uniform Zn^{2+} transportation. As comparison, due to the uneven deposition and the exist of side reactions, KHP-0 electrolyte shows an unstable interface with a continuously reduced Rct.

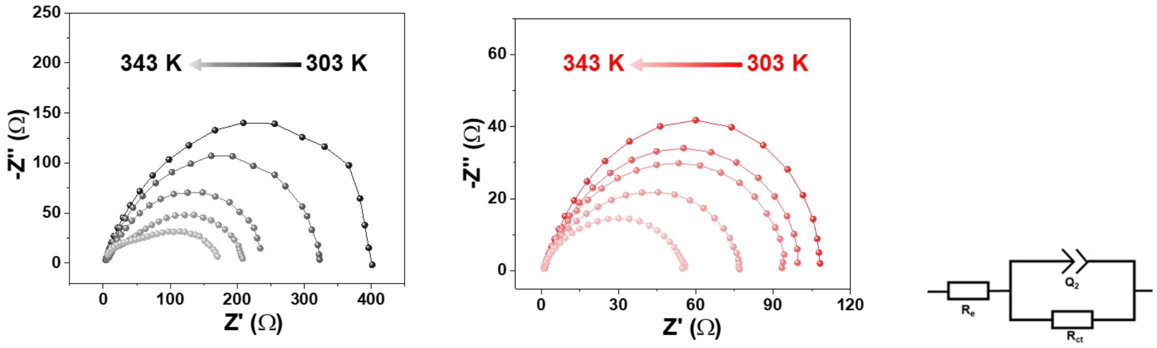

Figure S18. EIS curves under various temperatures in symmetrical batteries based on KHP-0 (left) and KHP-50 (right) electrolytes and corresponding fitted equivalent circuit.

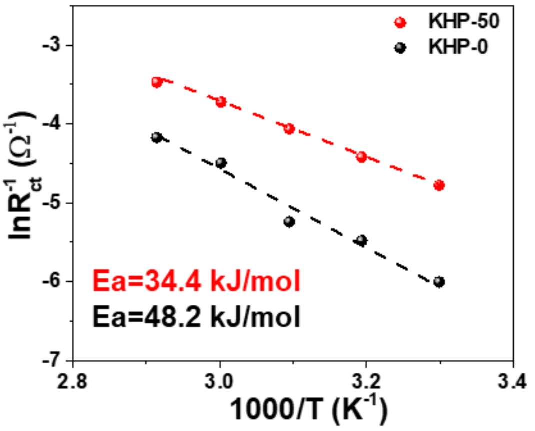

Figure S19. Activation energies of Zn^{2+} with different electrolytes calculated from EIS results.

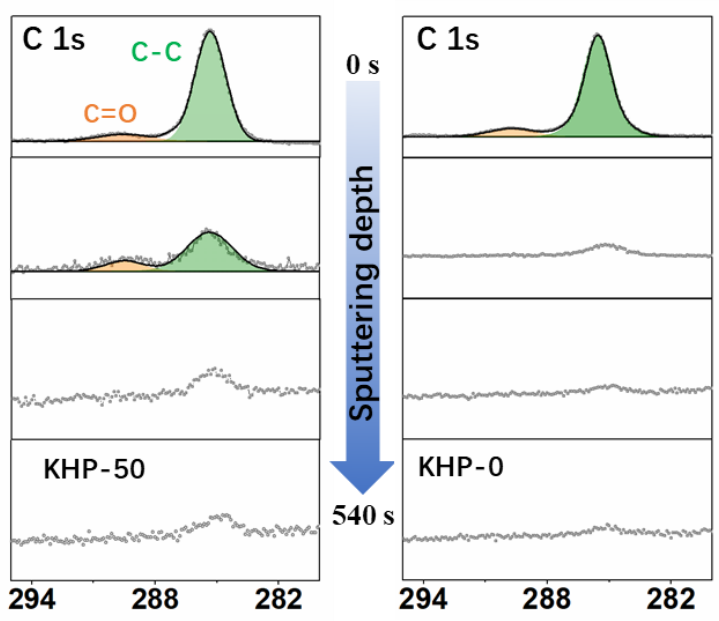

Figure S20. XPS spectral regions for C 1s with an argon (Ar+) sputtering depth for electrodes cycled in different electrolytes.

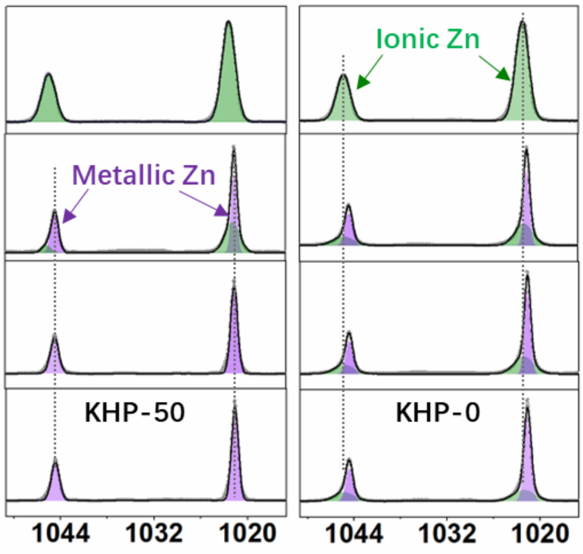

Figure S21. XPS spectra of Zn2p with an argon (Ar+) sputtering depth for electrodes cycled in different electrolytes.

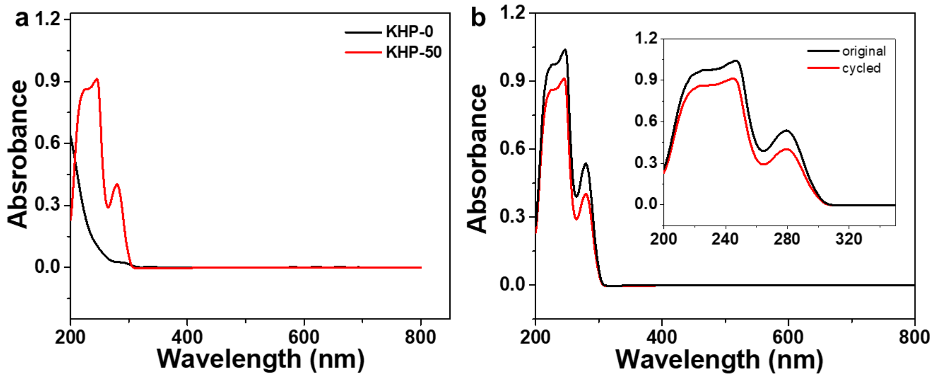

Figure S22. UV-vis spectroscopy of (a) KHP-0 and KHP-50 electrolyte, (b) KHP-50 electrolyte before and after cycling.

Note for S22: The charaterazition adsorbance of KHP additive is shown in Figure S22a. Due to the π-π* (bonding π orbital - antibonding π* orbital) and n-π* (non-banded orbital - antibonding π* orbital) transitions of C=C and C=O there are two obvious adsorption bands of HP^-: B band (210 nm ~ 250 nm)、and C band (260 nm ~ 300 nm).[5] Therefore, as shown in Figure S22b, the original KHP-50 electrolyte shows two broad peaks with the absorbance of 1.05 and 0.54 at 246.1 nm and 279.0 nm. After cycling, there is a decrease in absorbance for both peaks (0.91 at 245.9 nm and 0.40 at 278.8 nm), which indicating the concentration of HP^- decreased after cycling.

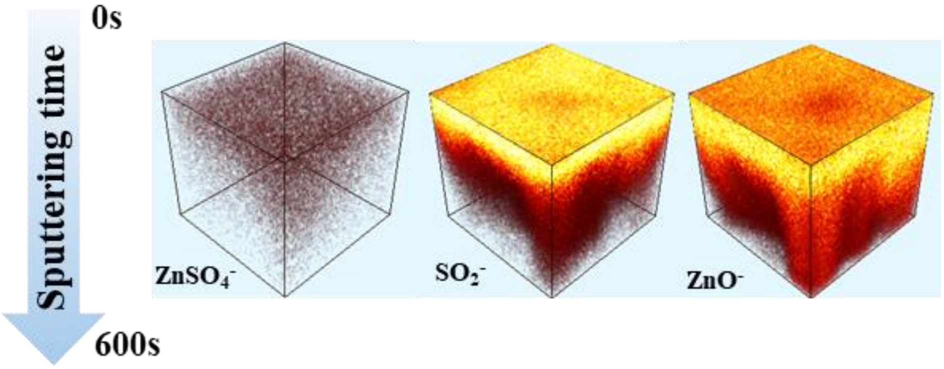

Figure S23. ToF-SIMS 3D view of $ZnSO_4^-$, SO_2^-, ZnO^- with gradually increasing argon (Cs^+) sputtering time for the electrode cycled in KHP-0 electrolyte.

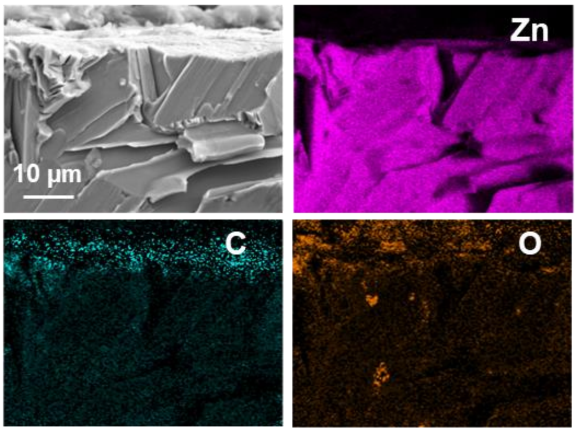

Figure S24. Cross-section SEM and EDS mapping of electrode cycled in KHP-50 electrolyte.

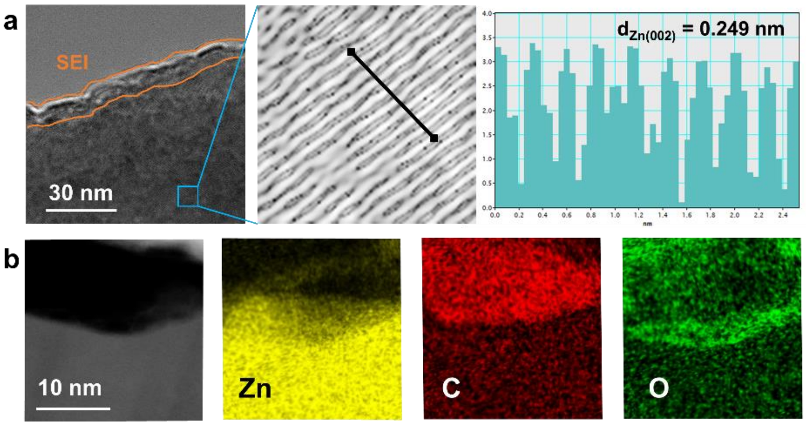

Figure S25. (a) TEM image with the corresponding inverse fast Fourier transform image and statistical line profile analysis for selected line, and (b) corresponding mapping with the elements of Zn, C, O for the electrode cycled in KHP-50 electrolyte.

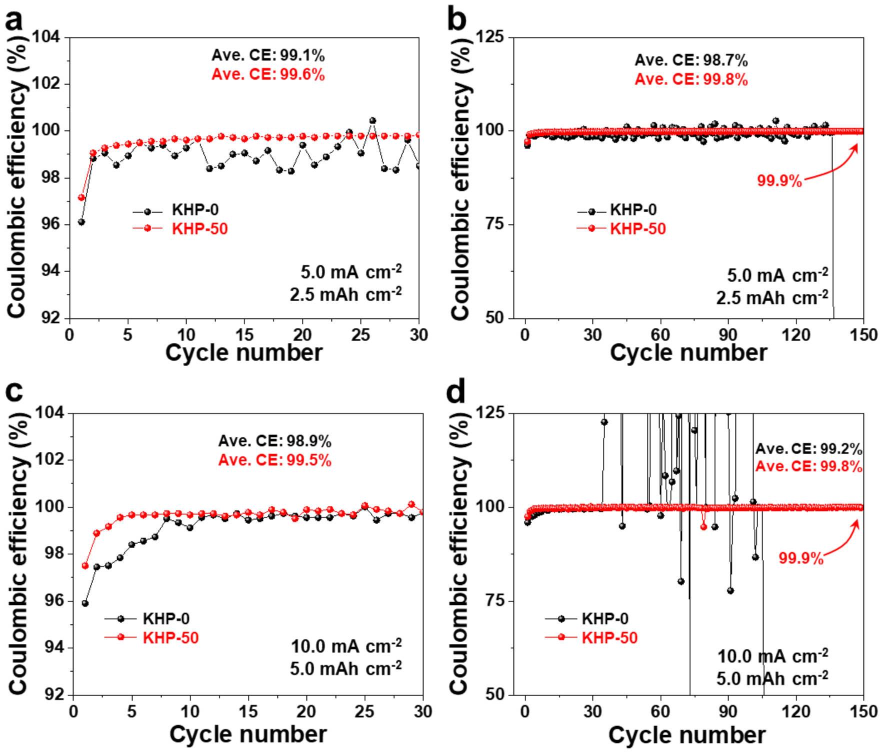

Figure S26. Coulombic efficiencies of different electrolytes under (a, b) 5.0 mA cm^{-2} and (c, d) 10.0 mA cm^{-2}.

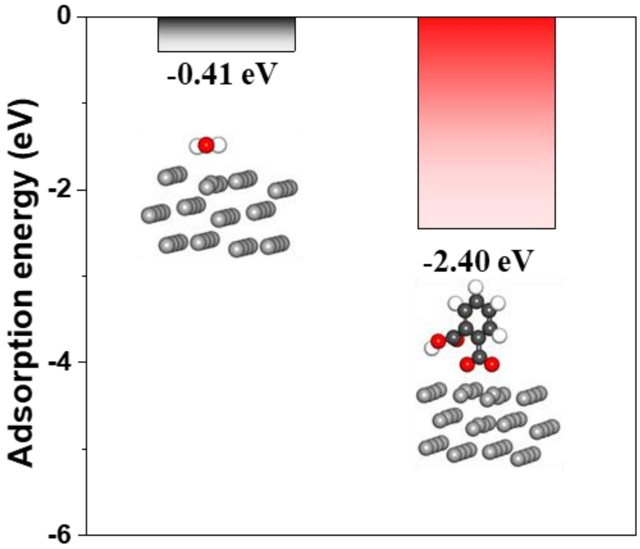

Figure S27. Adsorption energies of H_2O molecular and KHP additive on Zn (101) surface.

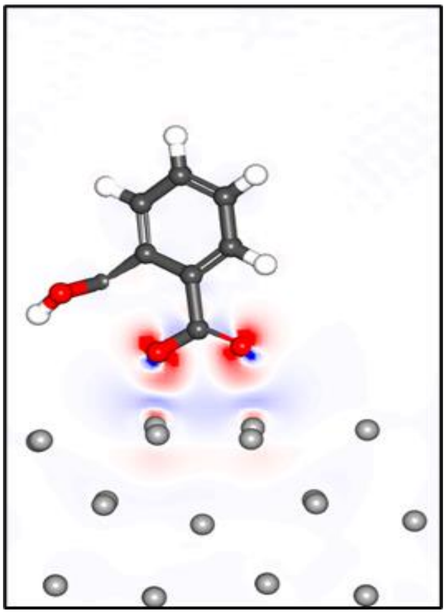

Figure S28. The electron density difference of Zn(101) adsorbed with KHP.

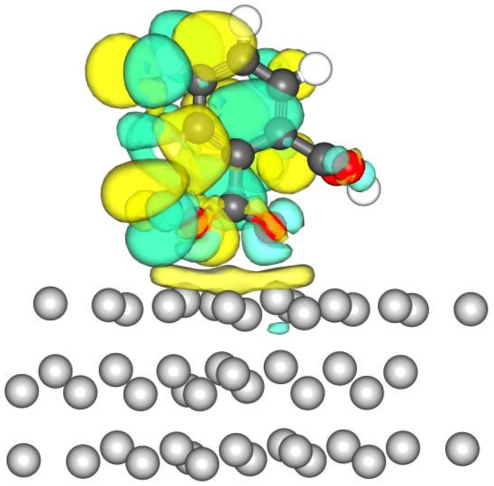

Figure S29. The electron density of Zn(101) adsorbed with KHP.

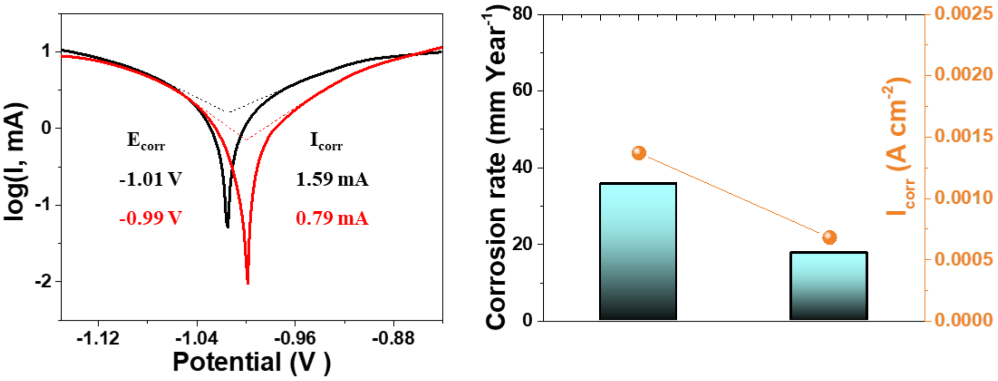

Figure S30. Tafel plots (left) and corresponding calculations (right) of the electrodes tested in KHP-0 and KHP-50 electrolytes.

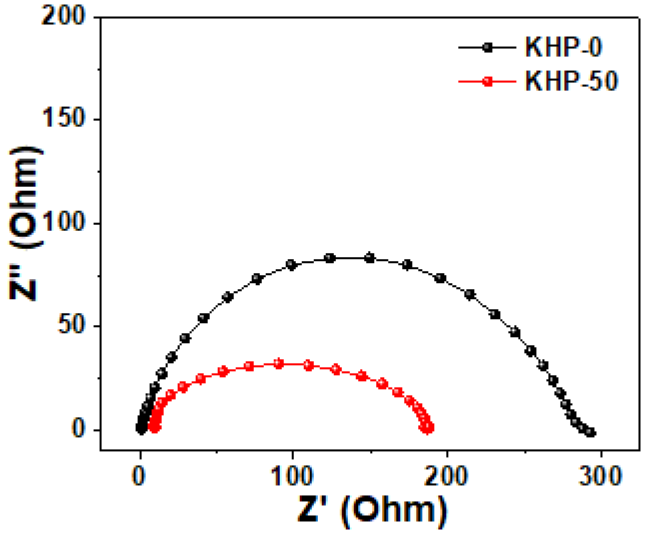

Figure S31. EIS curves of KHP-0 and KHP-50 electrolytes in symmetric cells under room temperature.

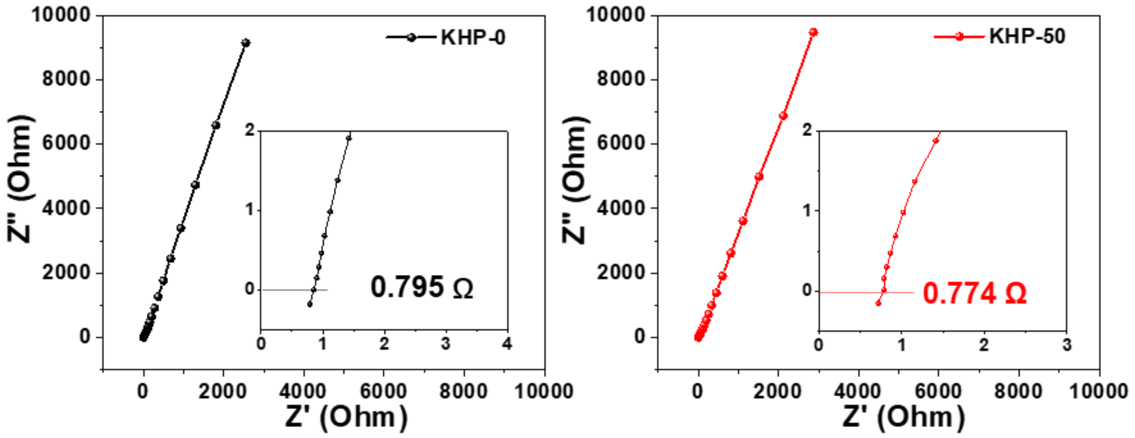

Figure S32. EIS measurements for ionic conductivity of KHP-0 and KHP-50 electrolytes under a freequency range of 300 kHz ~ 100 mHz.

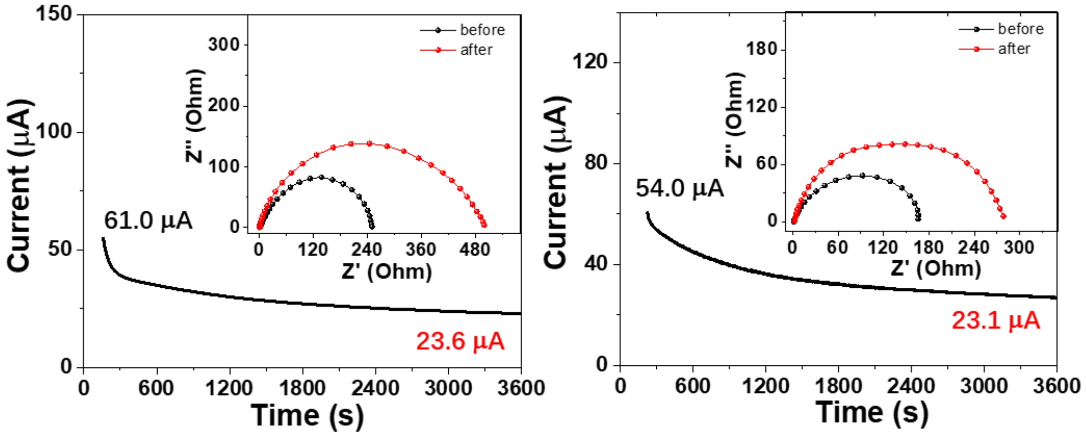

Figure S33. Current-time curves under constant voltage polarization of 20 mV and EIS results before and after polarization test in KHP-0 (left) and KHP-50 (right) electrolytes.

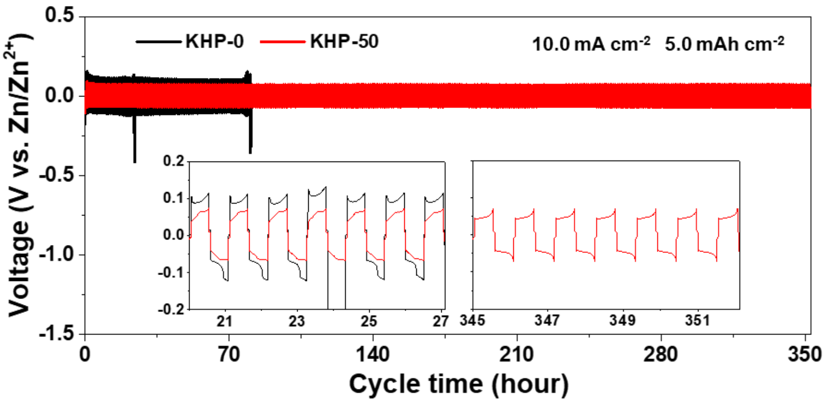

Figure S34. Symmetric cell of KHP-0 and KHP-50 electrolytes under 10.0 mA cm^{-2} with 5.0 mAh cm^{-2} (DOD: 43%).

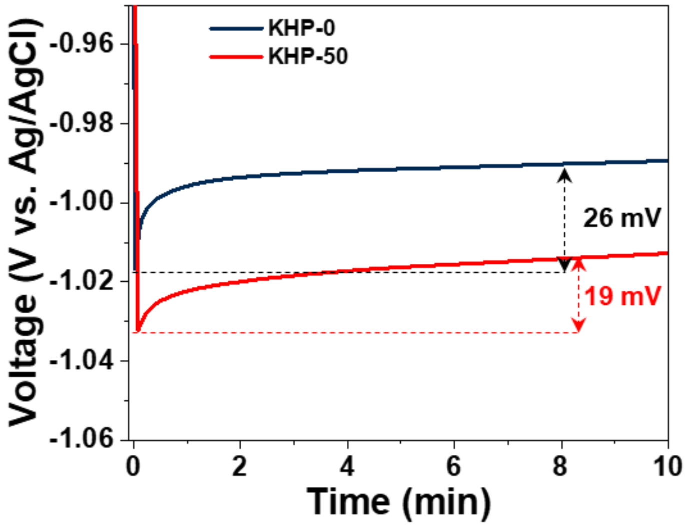

Figure S35. Nucleation overpotential of Zn^{2+} in different electrolytes.

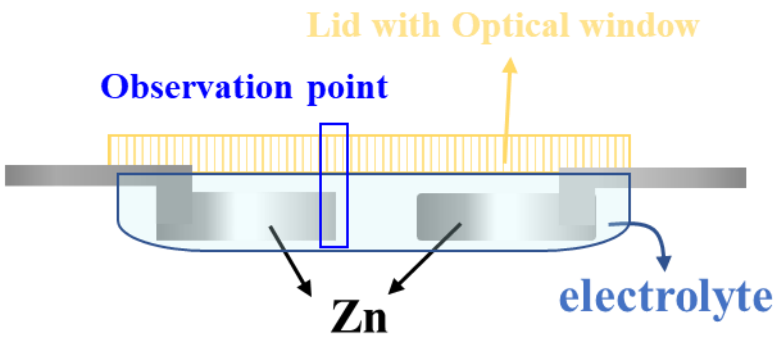

Figure S36. In-situ optical microscopy device for investigating the occurrence of morphological changes during Zn ion deposition.

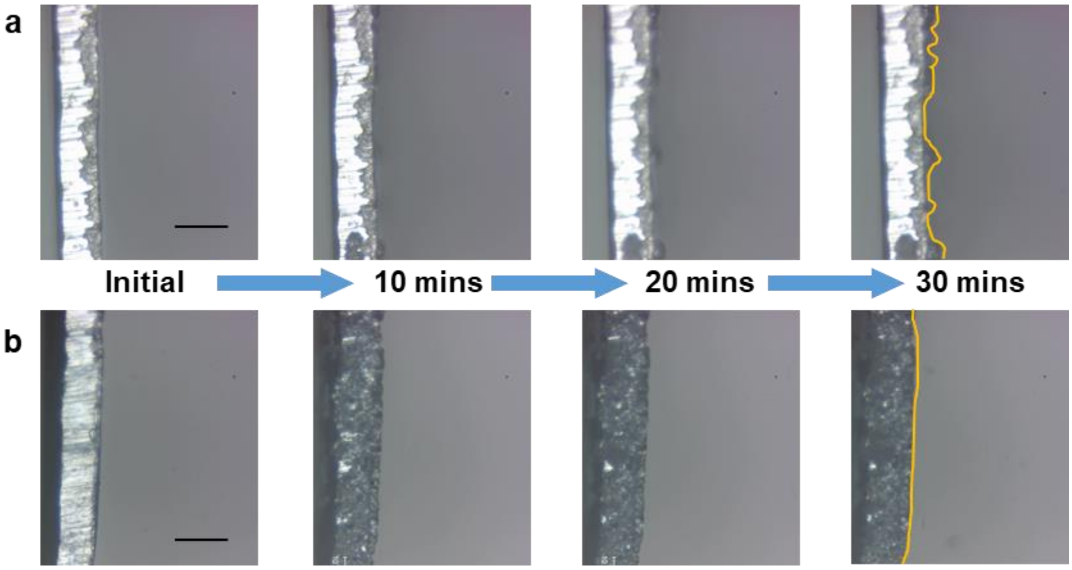

Figure S37. In-situ optical microscopy images of (a) KHP-0 and (b) KHP-50 electrolytes plating under 2 mA cm^{-2}. (scale bar: 30 um)

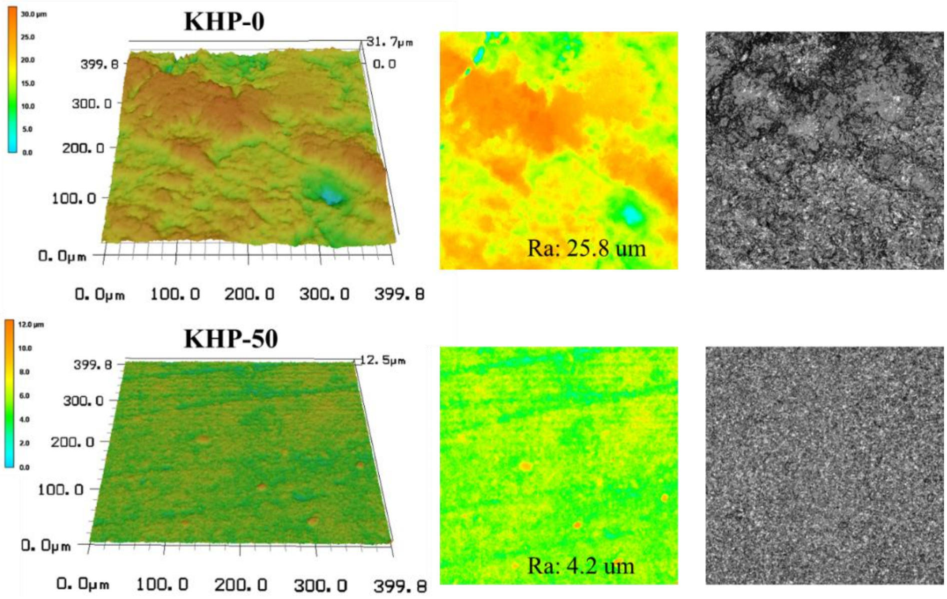

Figure S38. 3D confocal microscopy images of KHP-0 and KHP-50 electrolytes after plating at 2 mA cm^{-2}.

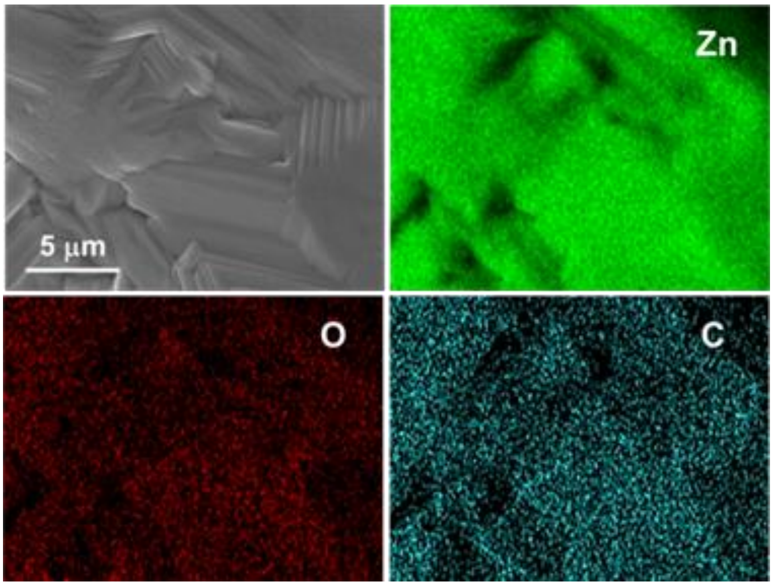

Figure S39. High resolution SEM images of Zn electrode cycled in KHP-50 electrolyte and corresponding EDS mapping.

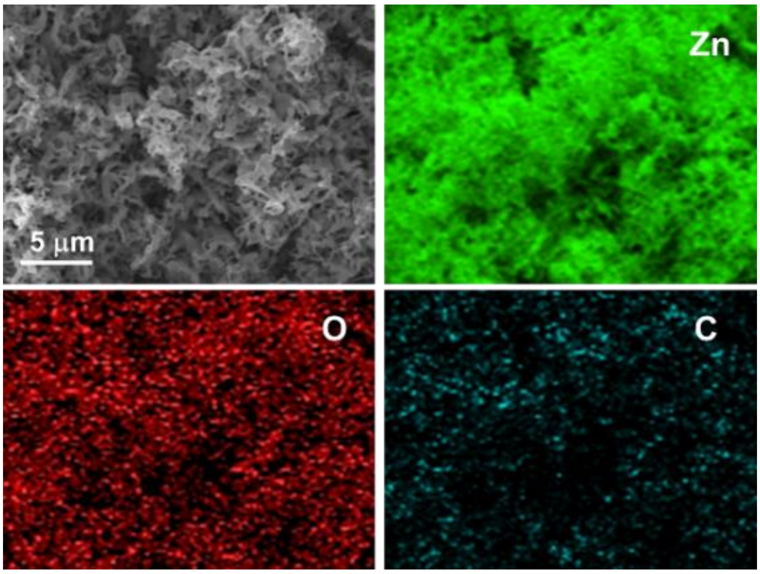

Figure S40. SEM images of Zn electrode cycled in KHP-0 electrolyte and corresponding EDS mapping.

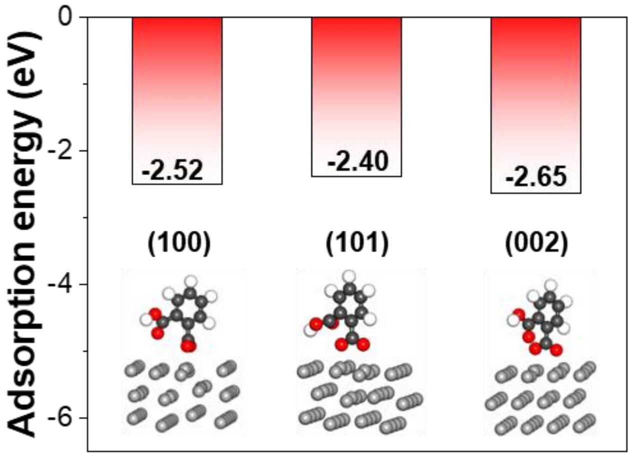

Figure S41. Adsorption energies of PH^- to different Zn crystal planes.

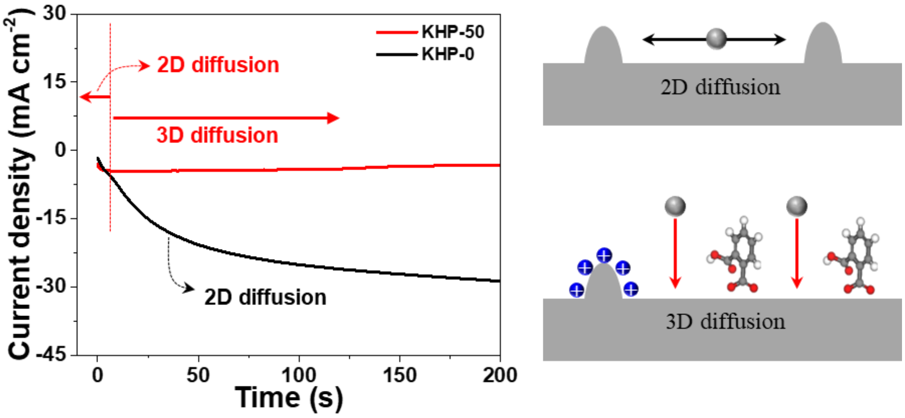

Figure S42. Chronoamperometric responses of KHP-0 and KHP-50 electrolytes under -150 mV in Zn symmetric cells.

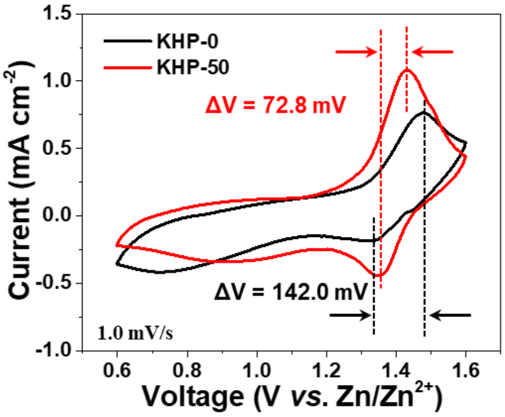

Figure S43. CV curves of KHP-0 and KHP-50 electrolytes under 1.0 mV/s in Zn||I_2 full cell.

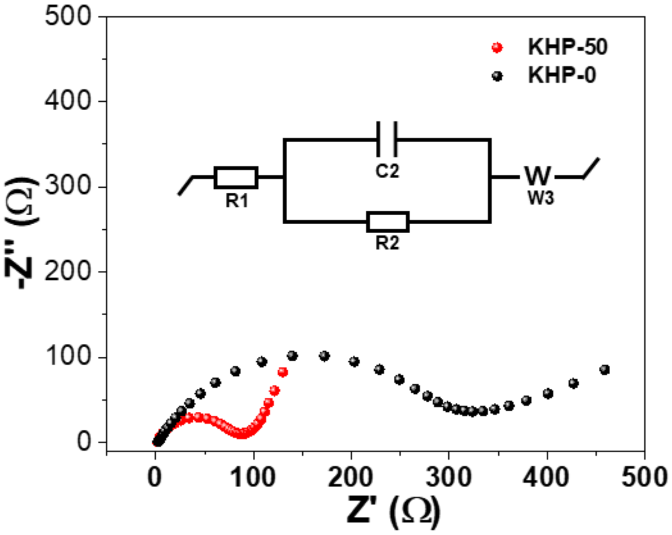

Figure S44. EIS plots of the full cell with KHP-0 and KHP-50 electrolytes after the 50th cycle.

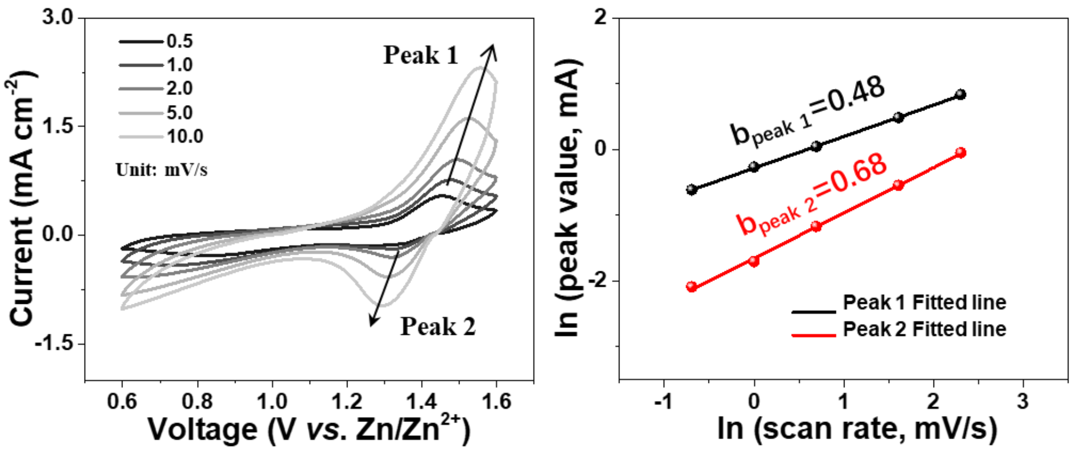

Figure S45. CV curves of KHP-0 electrolyte under different scan rates and corresponding b analysis of different peaks.

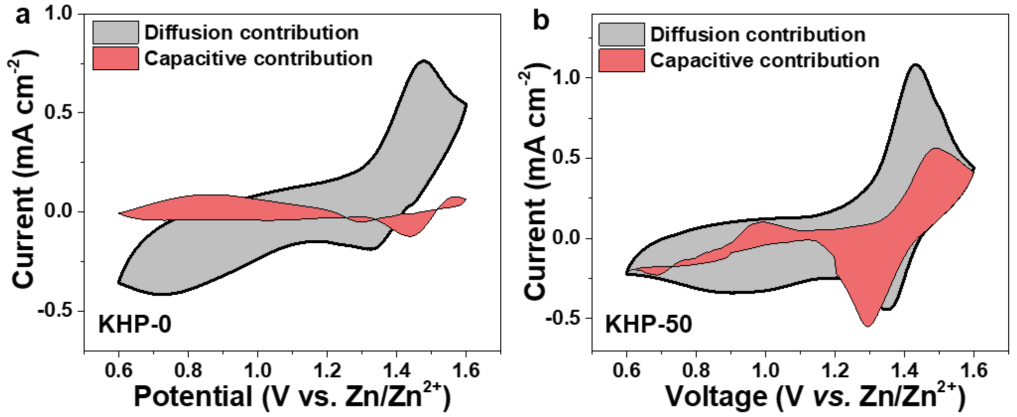

Figure S46. Surface Capacitive and diffusion process of Zn||I_2 full cells calculated from Figure 5c and S45.

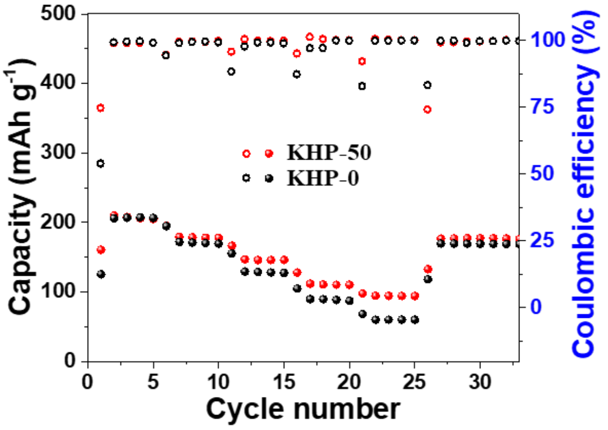

Figure S47. Rate performance of Zn-I_2 full cells in different electrolytes with free standing I_2 cathode.

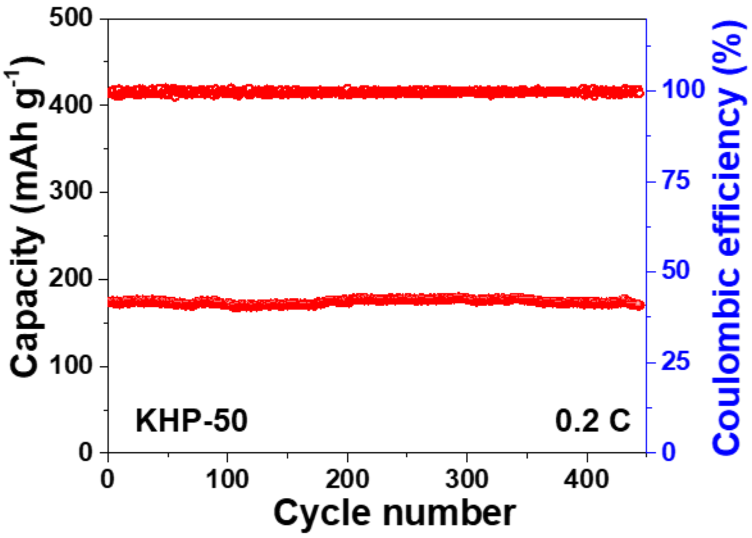

Figure S48. Cycle performance of Zn||I_2 full cell under 0.2 C after rate performance test.

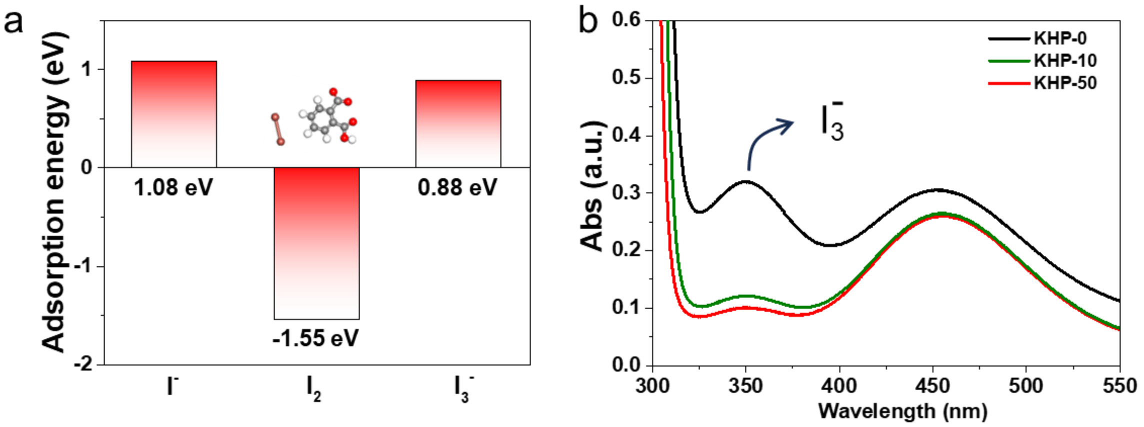

Figure S49. (a) Adsorption energy of HP anions toward I^-, I_2, and I_3^- species. (b) UV–vis spectra of iodine with different electrolytes.

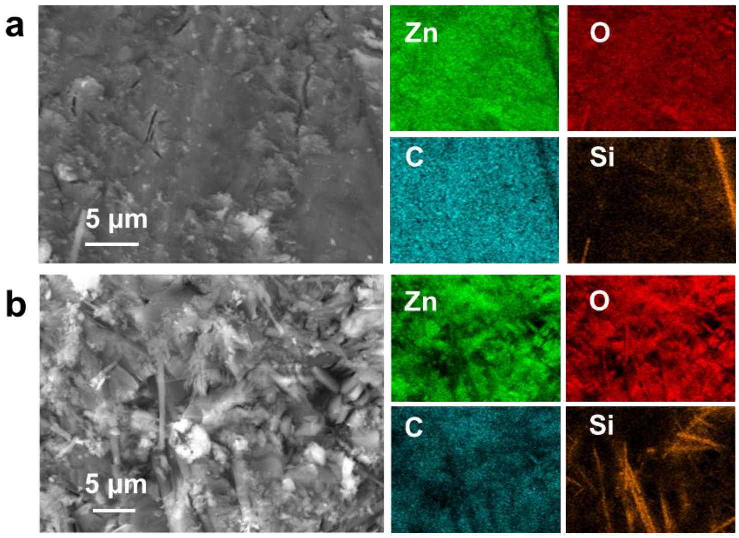

Figure S50. SEM images of Zn electrode after cycling in (a) KHP-50 (b) KHP-0 electrolytes.

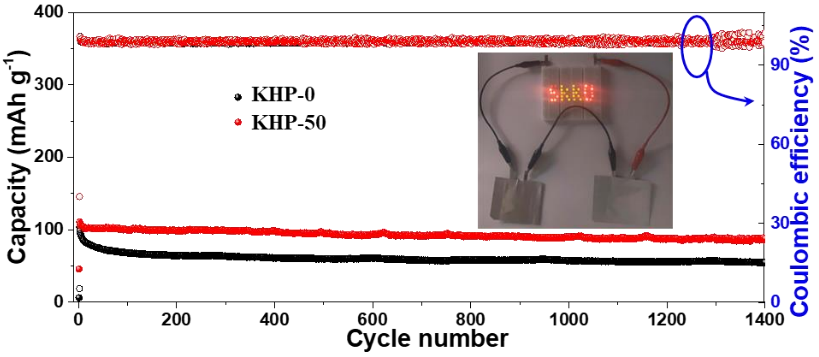

Figure S51. Cycle performance of Zn-I2 full cells in different electrolytes with free standing I2 catode.

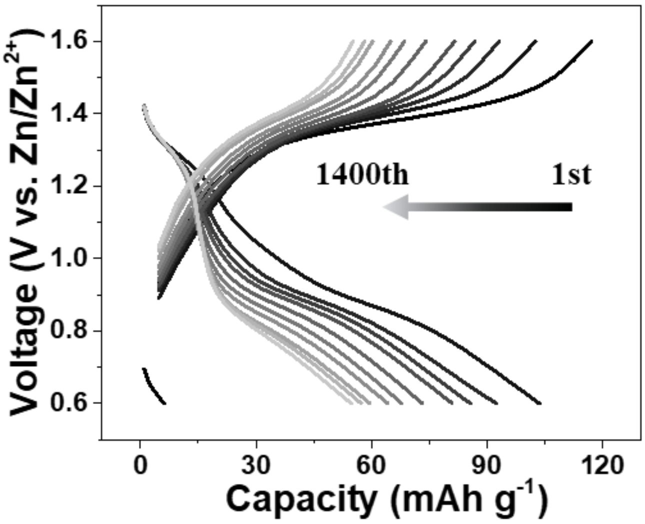

Figure S52. Voltage profiles of Zn||I2 full cell under 2.0 C in KHP-0 electrolyte.

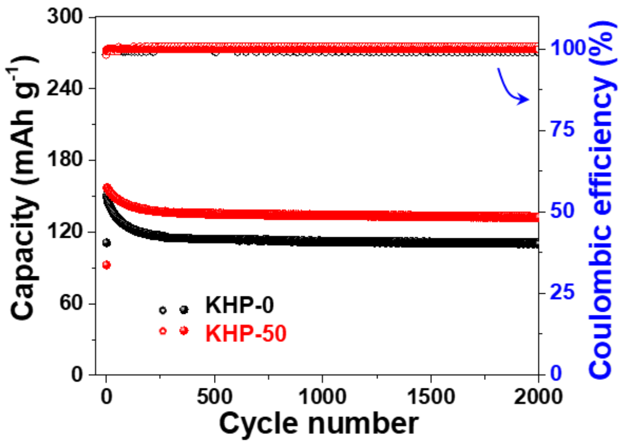

Figure S53. Cycle performance of Zn-I_2 full cells in different electrolyte.

IV. Supplementary tables

Table S1. The electrode potential of K^+ and Zn^{2+} with different concentrations.

Sample	E^{θ} (V)	$E_{2.0M}$(V)	$E_{0.1M}$(V)	$E_{0.05M}$(V)	$E_{0.01M}$(V)
Zn^{2+}	-0.762	-0.753	-	-	-
K^+	-2.931	-	-2.99	-3.01	-3.05

Table S2. Comparisons of the relative texture coefficient and relative crystallinity between the electrode cycled with/without K^+.

Sample	$I_{(002)}$	$I_{(100)}$	$I_{(101)}$	$RTC_{(002)}$	$RTC_{(100)}$	$RTC_{(101)}$
PDF	53	40	100	/	/	/
Without K^+	4256	3001	11562	29.64	27.69	42.67
With K^+	2532	1264	6530	33.02	21.85	45.14

Table S3. Tafel parameters, i.e., corrosion current and corrosion rate, calculated from Figure S6 and S30, of the electrodes cycled in $ZnSO_4$ with/without K^+ electrolytes.

Sample	E_{corr} (V)	I_{corr} (mA)	Corrosion rate (mm $year^{-1}$)
KHP-0	-1.01	1.59	35.7
KHP-0 with K^+	-1.02	1.38	31.0
KHP-50	-0.99	0.79	17.7

Table S4. The fitted results from the raman spectra at ~980 and ~390 cm^{-1}.

	KHP-0	KHP-10	KHP-50	KHP-100
**SSIP**	65.6%	79.2%	85.6%	89.6%
**Zn-OH$_2$**	41.5%	38.0%	35.1%	30.7%

Table S5. The pH values of the electrolytes during different cycle states.

	Initial	1st cycle	2nd cycle	5th cycle	10th cycle	10th - initial
**KHP-0**	3.76	4.41	4.61	4.93	5.32	1.56
**KHP-50**	3.34	4.02	4.07	4.19	4.37	1.03
**Difference (KHP-0)**	\	0.65	0.2	0.32	0.39	\
**Difference (KHP-50)**	\	0.68	0.05	0.12	0.18	\

Table S6. The solvation energy of KHP-50 electrolyte that considering the H_2O solvation process.

	[Zn·HP·n H_2O] (Ha)	[Zn·HP·(n-1) H_2O] (Ha)	H_2O (Ha)	Solvation energy (Ha)	Solvation energy (eV)
**n=5**	-2867.781229	-2787.27295	-80.472541	-0.037312	-0.972466718
**n=4**	-2787.269317	-2706.750121	-80.472385	-0.044088	-1.273768679
**n=3**	-2706.79015	-2626.267541	-80.472266	-0.072934	-1.369883373
**n=2**	-2626.268056	-2545.737518	-80.472615	-0.093994	-1.576140032
**n=1**	-2545.734281	-2465.196971	-80.472312	-0.140199	-1.768663299

Table S7. The solvation energy of KHP-50 electrolyte that considering the anion solvation process.

	[Zn·HP·n H_2O] (Ha)	[Zn· n H_2O] (Ha)	HP^- (Ha)	Solvation energy (Ha)	Solvation energy (eV)
**n=5**	-2867.781229	-2222.616422	-644.740442	-0.037312	-11.54739602
**n=4**	-2787.269317	-2142.084682	-644.742869	-0.044088	-12.02088918
**n=3**	-2706.787055	-2061.562434	-644.733763	-0.072934	-13.3567316
**n=2**	-2626.268056	-1981.003285	-644.73131	-0.093994	-14.51600455
**n=1**	-2545.734281	-1900.412914	-644.727087	-0.140199	-16.1709558

Table S8. The solvation energy of KHP-0 electrolyte with different solvation structures.

	[Zn·n H_2O] (Ha)	[Zn·(n-1) H_2O] (Ha)	H_2O (Ha)	Solvation energy (Ha)	Solvation energy (eV)
**n=6**	-2303.164725	-2222.655028	-80.472385	-0.037312	-1.015296832
**n=5**	-2222.655028	-2142.138555	-80.472385	-0.044088	-1.199678568
**n=4**	-2142.138555	-2061.593236	-80.472385	-0.072934	-1.984607074
**n=3**	-2061.593236	-1981.026857	-80.472385	-0.093994	-2.557670734
**n=2**	-1981.026857	-1900.414273	-80.472385	-0.140199	-3.814954989

Table S9. Fitted R_{ct} of Zn symmetric cell with KHP-0 electrolyte at various temperatures, calculated from Figure S18.

Sample	T (K)	R_{ct} (Ω)	$ln(R_{ct})^{-1}$	Error (%)
	303.15	405.3	-6.01	4.13
	313.15	239.8	-5.48	2.20
**KHP-0**	323.15	189.0	-5.24	3.52
	333.15	89.8	-4.50	2.21
	343.15	65.0	-4.17	3.61

Table S10. Fitted R_{ct} of Zn symmetric cell with KHP-50 electrolyte at various temperatures, calculated from Figure S18.

Sample	T (K)	R_{ct} (Ω)	$ln(R_{ct})^{-1}$	Error (%)
	303.15	118.9	-4.78	3.08
	313.15	83.3	-4.42	2.09
**KHP-50**	323.15	58.3	-4.07	4.21
	333.15	41.4	-3.72	1.77
	343.15	32.3	-3.47	2.36

Table S11. SEM-EDS elemental analysis of Figure S24.

Element	Wt%	At%
Zn	83.93	50.2
O	3.1	7.5
C	12.97	42.3

Table S12. Fitted R_{ct} (charge transfer resistance) of symmetric cells, calculated from Figure S31.

Sample	R_{ct} (Ω)	Error (%)
**KHP-0**	272.5	7.02
**KHP-50**	154.6	6.14

Table S13. Fitted R_{ct} (charge transfer resistance) of symmetric cells, calculated from Figure S33.

Sample	R_{ct} (Ω)	Error (%)
**KHP-0**	230.5	4.33
**KHP-0**	525.8	1.17
**KHP-50**	169.3	5.80
**KHP-50**	224.9	4.30

Table S14. The comparison of symmetric cells between recently reported electrolyte additive works and our work.

Electrolytes additives	Current density (mA cm^{-2})	Discharge capacity (mAh cm^{-2})	Lifespan (h)	Overpotential (mV)	Accumulative capacity (mAh cm^{-2})	Ref.
Octyltrimethyl ammonium bromide	5.0	1.0	2500	~95	2500	[68]
3-(hydroxy(phenyl)phosphoryl) propanoic acid	0.5	0.5	3000	100	1500	[69]
BmimCl	2.0	2.0	2000	~50	4000	[70]
Triethyl phosphate	5.0	5.0	~660	over 100	3300	[71]
N-Acetyl-ε-caprolactam	0.2	0.2	9800	~45	1960	[72]
Disodium succinate	1	1	2300	70	2300	[73]
Gallium nitrate	1.2	0.6	4000	20	2400	[74]
**potassium hydrogen phthalate**	**5.0**	**2.5**	**2600**	**28.2**	**6500**	**This work**

Table S15. SEM-EDS elemental analysis of Figure S39.

Element	Wt%	At%
Zn	86.90	55.59
O	1.42	3.72
C	11.68	40.69

Table S16. SEM-EDS elemental analysis of Figure S40.

Element	Wt%	At%
Zn	90.99	65.89
O	5.40	20.13
C	3.61	13.98

Table S17. Comparisons of the relative texture coefficient between the electrode cycled in KHP-0 and KHP-50.

Sample	$I_{(002)}$	$I_{(100)}$	$I_{(101)}$	$RTC_{(002)}$	$RTC_{(100)}$	$RTC_{(101)}$
PDF	53	40	100	/	/	/
KHP-0	33.67	29.38	63.71	31.66	36.60	31.75
KHP-50	33.52	33.08	65.98	31.52	41.21	32.88

Table S18. Fitted EIS date of full cells from Figure S44.

Sample	R_1 (Ω)	R_2 (Ω)	Error (%)
**KHP-0**	2.832	217.0	3.77
**KHP-50**	2.198	68.3	1.34

Table S19. The comparison of performance in Zn-I_2 full cells between recently reported electrolyte additive works and our work.

Electrolytes additives	Current density (C)	Capacity (mAh g^{-1})	Lifespan (h)	Capacity retention	Ref.
Erythritol/ taurine	5.0	133	5000	75%	[6]
cysteamine	9.5	121.3	10000	88%	[7]
silk protein	2.3	164	1000	/	[8]
Succinamic acid	4.7	77.3	400	75.4%	[9]
pyridine /imidazole	47.4	105.5	25000	/	[10]
NiTi@Zn	23.7	83	20000	61.4%	[11]
**potassium hydrogen phthalate**	**10.0**<br>**50.0**	**133.2**<br>**113.8**	**2000**<br>**8800**	**85.0%**	**This work**

References

[1] F. X. Wang; Z. C. Liu; C. Q. Yang; H. X. Zhong; G. Nam; P. P. Zhang; R. H. Dong; Y. P. Wu; J. Cho; J. Zhang; X. L. Feng, *Adv. Mater.* **2020**, 32, 1905361.

[2] H. J. Tian; T. Gao; X. G. Li; X. W. Wang; C. Luo; X. L. Fan; C. Y. Yang; L. M. Suo; Z. H. Ma; W. Q. Han; C. S. Wang, *Nat. Commun.* **2017**, 8, 14083.

[3] S. Y. Huang, P. Zhang, J. Lu, J. S. Kim, D. H. Min, J. S. Byun, M. J. Kim, H. Fu, P. X. Xiong, P. J. Yoo, W. W. Li, X. Yu, X, Qin, H. S. Park, *Energy Environ. Sci.*, **2024**, (DOI:10.1039/D4EE02867H)

[4] L. D. Xing; X. W. Zheng; M. Schroeder; J. Alvarado; A. V. Cresce; K. Xu; Q. S. Li; W. S. Li, *Accounts Chem. Res.* **2018,** *51*, 282-289.

[5] N. V. Karimova, M. Luo, V. H. Grassian, R. B. Gerber, *Phys. Chem. Chem. Phys.*, **2020**, 22(9): 5046-5056

[6] R. Wang, Z. Liu, J. Wan, X. Zhang, D. Xu, W. Pan, L. Zhang, H. Li, C. Zhang, Q. Zhang, *Adv. Energy Mater.*, **2024,** 2402900. (doi.org/10.1002/aenm.202402900)

[7] Z. Wei, S. Wang, D. Li, S. Yang, S. Guo, G. Qu, Y. Yang, H. Li, *Energy Environ. Sci.*, **2024,** (doi.org/10.1039/D4EE01260G)

[8] S. J. Zhang, J. Hao, H. Wu, Q. Chen, C. Ye, S. Z. Qiao, *Adv. Mater.*, **2024,** 36(35): 2404011.

[9] Q. T. Chen; K. F. Ouyang; Y. Y. Wang; M. F. Chen; H. W. Mi; J. Z. Chen; C. X. He; H. Li; D. T. Ma; P. X. Zhang, *Adv. Funct. Mater.* **2024,** 2406386.

[10] Y. Lyu; J. A. Yuwono; P. T. Wang; Y. Y. Wang; F. H. Yang; S. L. Liu; S. L. Zhang; B. F. Wang; K. Davey; J. F. Mao; Z. P. Guo, *Angew. Chem. Int. Edit.* **2023,** *62*, 2303011.

[11] K. K. Sonigara; J. V. Vaghasiya; M. Pumera, Adv. Energy Mater. 2024, 2401321.
